# Mathematical epidemiological modeling and analysis of monkeypox dynamism with non-pharmaceutical intervention using real data from United Kingdom

**DOI:** 10.3389/fpubh.2023.1101436

**Published:** 2023-02-17

**Authors:** Mercy Ngungu, Emmanuel Addai, Adejimi Adeniji, Umar Muhammad Adam, Kayode Oshinubi

**Affiliations:** ^1^Human Sciences Research Council (HSRC), Pretoria, South Africa; ^2^Department of Biomedical Engineering, College of Biomedical Engineering, Taiyuan University of Technology, Taiyuan, China; ^3^Department of Mathematics, Taiyuan University of Technology, Taiyuan, China; ^4^Department of Mathematics, Tshwane University of Technology, Pretoria, South Africa; ^5^Department of Mathematics, Federal University, Dutse, Nigeria; ^6^AGEIS Laboratory, University Grenoble Alpes, Saint Martin d'Hères, France

**Keywords:** Caputo-Fabrizio fractional derivative, reproduction number, parameter estimation, numerical scheme, data fitting

## Abstract

In this study, a mathematical model for studying the dynamics of monkeypox virus transmission with non-pharmaceutical intervention is created, examined, and simulated using real-time data. Positiveness, invariance, and boundedness of the solutions are thus examined as fundamental features of mathematical models. The equilibrium points and the prerequisites for their stability are achieved. The basic reproduction number and thus the virus transmission coefficient ℜ_0_ were determined and quantitatively used to study the global stability of the model's steady state. Furthermore, this study considered the sensitivity analysis of the parameters according to ℜ_0_. The most sensitive variables that are important for infection control are determined using the normalized forward sensitivity index. Data from the United Kingdom collected between May and August 2022, which also aid in demonstrating the usefulness and practical application of the model to the spread of the disease in the United Kingdom, were used. In addition, using the Caputo–Fabrizio operator, Krasnoselskii's fixed point theorem has been used to analyze the existence and uniqueness of the solutions to the suggested model. The numerical simulations are presented to assess the system dynamic behavior. More vulnerability was observed when monkeypox virus cases first appeared recently as a result of numerical calculations. We advise the policymakers to consider these elements to control monkeypox transmission. Based on these findings, we hypothesized that another control parameter could be the memory index or fractional order.

## 1. Introduction

The unexpected breakout and global spread of monkeypox have drawn the attention of scientists due to the continuing COVID-19 pandemic. The prevalence of the largest and most pervasive monkeypox pandemic outside of Africa as of 22 June 2022, is 3,340 confirmed cases reported across the world. In addition to mother-to-child vertical transmission, the monkeypox virus can spread from person to person by direct contact with infectious skin or mucosal skin lesions, respiratory droplets, or indirect contact with contaminated objects or materials. The possibility of community transmission cannot be ruled out, and it may also be sexually transferred by semen or vaginal fluid. The virus that causes monkeypox is called the monkeypox virus, and it is an enveloped, linear, double-stranded DNA virus that belongs to the Chordopoxvirinae subfamily of the Poxviridae family. With symptoms of the disease lasting 2–4 weeks and a death rate that previously ranged from 0 to 11 deaths, monkeypox is often a self-limiting sickness. Intense headaches, fever, lesions, and lymphadenopathy are some of the symptoms of monkeypox. Antiviral medications and smallpox vaccines have been approved for use in various nations in response to the monkeypox outbreak, despite the fact that there is no specific treatment or vaccine for monkeypox virus infection. Before allowing the virus to successfully establish person-to-person transmission, quick action is required to stop the local development of the disease and, consequently, the global monkeypox outbreak (1–11). In Peter et al. (12), modeling and optimal control were used to study monkeypox and the cost-effective strategies were investigated. This study shows that, among all competing measures, combining preventative measures to reduce rodent-to-human disease transmission is the most practical and cost-effective option.

Numerous research articles have been published where both classical and fractional models were constructed, and there is a plethora of literature on modeling infectious diseases. Because fractional-order derivative has unique properties such as heredity and memory that enable it to fully comprehend the dynamics of real phenomena, an analysis based on fractional-order derivative is more advantageous and practical than an analysis based on classical derivative (13, 14). At two separate closed locations, the phenomenon is indistinguishable by the standard derivatives. A generalized derivative known as the fractional order was proposed to address the problems with ordinary derivatives (15). Many researchers used fractional- order derivatives in many fields, as shown in Kumar et al. (16), Higazy et al. (17), Djida and Atangana (18), Baba (19), Owolabi and Atangana (20), Mohammadi et al. (21), Baleanu et al. (22), and Wutiphol and Turab (23). In the realm of mathematical biology, the Mittag–Leffler-type kernel has been used continuously over other derivatives, and numerous epidemiological models, such as for dengue fever, smoking, tuberculosis, measles, Ebola, and other diseases, have been studied using this operator as shown in Asamoah et al. (24), Peter et al. (25, 26), Kumar et al. (27), Morales-Delgadoa et al. (28), Atangana and Baleanu (29), and Atangana et al. (30). Most notably, in Zhang et al. (31), the Mittag–Leffler-type kernel modeling for Ebola–malaria co-infection was investigated by the authors with the best possible control. They strongly recommended the Mittag–Leffler-type kernel. In Kumar et al. (32), investigated the COVID-19 model using singular and non-singular fractional operators and compared the results of these operators. In Aslam et al. (33), the authors examined a recent study on the mathematical modeling of HIV/AIDS using the Mittag–Leffler-type kernel and came to the conclusion that the infection rate decreases with decreasing operator. In Evirgen (34), the authors studied the transmission dynamics of the Nipah virus using the Caputo derivative. One of the interesting segments of their study was to focus on tracing the influence of fractional-order derivatives on the manner in which the model responds. In Ucar (35), the authors investigated a fractional SAIDR model within the framework of the Mittag–Leffler-type kernel. The effectiveness of the fractional operator is shown through a numerical simulation.

Considering the characteristics of exponential decay, the Caputo–Fabrizio fractional-order operator has been preferred over Atangana–Beleanu beta derivatives and a few other operators in the field of mathematical biology with more information (17–19, 24, 36–39). For instance, in Addai et al. (40), the authors studied a novel model of COVID-19 incorporating Alzheimer's disease using the Caputo–Fabrizio fractional-order operator. The results of the aforementioned study revealed that the two diseases have a link and the authors also concluded that the fractional operator is related to the rate of infection. In Shaikh and Nisar (41), the authors also considered the transmission dynamics of a fractional-order typhoid fever model using the Caputo–Fabrizio operator and the existence theory and achieved numerical solutions. In Shah et al. (42), Shah and his co-authors conducted a semi-analytical study of the Pine Wilt Disease (PWD) model with a convex rate *via* fractional order involving a non-singular kernel. To comprehend the trade-off between the lockdown and the transmission of the virus, Ahmed and his co-authors devised a five-term dynamical system (43). Another use of the Caputo–Fabrizio fractional-order operator was indicated, for instance, in Addai et al. (40), Shaikh and Nisar (41), Shah et al. (42), Ahmed et al. (43), Ullah et al. (44), Abboubakar et al. (45).

Furthermore, in Peter et al. (46), the authors used real data from Nigeria to study the dynamics of the transmission of the monkeypox virus using fractional calculus. The authors presented an argument on the modeling system by studying the infection control policies that will help the public to better understand the significance of control parameters in the eradication of the virus in the studied population. Furthermore, the transmission dynamics of the monkeypox virus was studied using a mathematical modeling approach in Peter et al. (47). In their findings, the authors indicated that the isolation of infected individuals in the human population helps reduce the transmission of the disease, which can serve as a form of intervention to control the spread of the virus.

We observed that none of the studies on the monkeypox virus and its modes of transmission took into account the interaction between the isolated and exposed compartments in the human subpopulation and the results of that contact rate with the rodent population and applied the modeling approach to real data from the United Kingdom. The major goals of this research are to calculate the exponential growth rate of the monkeypox virus, to forecast what might occur in future and how to stop it from spreading, and to understand the effects of non-pharmaceutical intervention on infected individuals, which will be able to guide us on how to deploy intervention resources to contain the spread of the disease. The remaining sections of the article are structured as follows: Section 2 presents some basic definitions and preliminary information, Section 3 presents the model formulation, Sections 4 deals with the dynamism of the model, Section 5 computes the basic reproduction number and some basic mathematical analysis, Section 6 present the endemic equilibrium of the model, Section 7 proves the existence and uniqueness of our model, Section 8 deals with the fitting of the model to real data from the United Kingdom, Section 9 presents numerical schemes and numerical simulations, Section 10 deals with sensitivity analysis, and Section 11 provides some perspectives, discussion, and conclusion.

## 2. Preliminaries

In this section, we review several key definitions, lemmas, and concepts that are necessary to understand the suggested model.

**Definition 2.1** Let *f* ∈ *Q*^1^(*p, q*), *q* > *p*, and α_∗_ ∈ (0, 1) (17), (40). Then, the Caputo–Fabrizio fractional-order derivative can be defined as

$${}_{p}^{CF}D_{t}^{\alpha }f\left(t\right)=\frac{G\left(\alpha \right)}{1-\alpha }\int_{p}^{t}f^{\prime }\left(x\right)exp\left[-\alpha \frac{t-s}{1-\alpha }\right]ds.$$

Here, *G*(α) is a normalization function, where *G*(0) = *G*(1) = 1. The fractional integral of the Caputo–Fabrizio fractional order is defined by:

$$I_{t}^{\alpha }f\left(t\right)=\frac{2\left(1-\alpha \right)}{2\left(1-\alpha \right)G\left(\alpha \right)}f\left(t\right)+\frac{2\alpha }{\left(2-\alpha \right)G\left(\alpha \right)}\int_{0}^{t}f\left(s\right)ds,t\geq 0.$$

**Lemma 2.2** Assuming there is a function *u*(*t*) ∈ *W*_*l*_[0, η], then the solution of fractional differential equation

$$\begin{array}{l} \{\begin{array}{l} {}^{CF}D_{t}^{\alpha }f\left(t\right)=u\left(t\right),t\in \left[0,\eta \right],\\ f\left(0\right)=f_{0}, \end{array} \end{array}$$

is given by

$$f\left(t\right)=f_{0}+\frac{2\left(1-\alpha \right)}{2\left(1-\alpha \right)G\left(t\right)}f\left(t\right)+\frac{2\alpha }{\left(2-\alpha \right)G\left(\alpha \right)}\int_{0}^{t}f\left(s\right)ds,t\geq 0$$

(24),(17), (40).

**Lemma 2.3** Suppose ***A*** ⊂ ***B*** be a closed convex non-empty subset of ***A*** and there exist two operators, *T*_1_ and *T*_2_, then it is Krasnoselskii's fixed point theorem (40) and it follows that:

- *T*_1_*u* + *T*_1_*u* ∈ ***A***, ∀*u* ∈ ***A***;
- *T*_1_ is contraction and *T*_2_ continuous and compact. Then quantify at least one solution *u* ∈ ***A*** such that
  $$\;T_{1}u+T_{2}u=u.$$

## 3. Model formulation

Using a system of differential equations, we studied both human and rodent populations in a closed homogeneous environment. There are five compartments in a human population of size *N*_*h*_(*t*): Susceptible *S*_*h*_(*t*); Exposed *E*_*h*_(*t*); Infected *I*_*h*_(*t*); Isolation/Quarantine *Q*_*h*_(*t*); and Recovered *R*_*h*_(*t*); where *N*_*h*_(*t*) = *S*_*h*_(*t*) + *E*_*h*_(*t*) + *I*_*h*_(*t*) + *Q*_*h*_(*t*) + *R*_*h*_(*t*). The rodent population *N*_*r*_(*t*) is split into *S*_*r*_(*t*) Susceptible; *E*_*r*_(*t*) Exposed; and *I*_*r*_(*t*) Infected. Let *N*_*r*_(*t*) = *S*_*r*_(*t*) + *E*_*r*_(*t*) + *I*_*r*_(*t*). From the aforementioned description, using the ideas in Yinka-Ogunleye et al. (5), we extend the studies of Peter et al. (46) and (47), then the ordinary differential equations in system (1) describe the dynamics of monkeypox transmission incorporating non-pharmaceutical intervention;

(1) $$\begin{array}{l} \{\begin{array}{l} \frac{dS_{h}}{dt}=\Lambda_{h}+\xi_{h}R_{h}+\theta_{h}Q_{h}-\lambda_{h}S_{h}-\mu_{h}S_{h},\\ \frac{dE_{h}}{dt}=\lambda_{h}S_{h}-\gamma_{h}E_{h}-\phi_{h}E_{h}-\mu_{h}E_{h},\\ \frac{dI_{h}}{dt}=\phi_{h}E_{h}-\left(\psi_{h}+\mu_{h}+\nu_{h}\right)I_{h},\\ \frac{dQ_{h}}{dt}=\gamma_{h}E_{h}-\left(\theta_{h}+\delta_{h}+\mu_{h}+\nu_{h}\right)Q_{h},\\ \frac{dR_{h}}{dt}=\psi_{h}I_{h}+\delta_{h}Q_{h}-\xi_{h}R_{h}-\mu_{h}R_{h},\\ \frac{dS_{r}}{dt}=\Lambda_{r}-\lambda_{r}S_{r}-\mu_{r}S_{r}\\ \frac{dE_{r}}{dt}=\lambda_{r}S_{r}-\phi_{r}E_{r}-\mu_{r}E_{r},\\ \frac{dI_{r}}{dt}=\phi_{r}E_{r}-\left(\mu_{r}+\nu_{r}\right)I_{r}, \end{array} \end{array}$$

where $\lambda_{h}=\frac{\beta_{rh}I_{r}+\beta_{hh}I_{h}}{N_{h}},\;\lambda_{r}=\frac{\beta_{rr}I_{r}}{N_{r}}$. To capture the memory in the predictions of the monkeypox virus transmission model and also to verify that both sides of the fractional equations have exact dimensions, the time-dependent kernel is defined by the power law correlation function, as in Tilahuna et al. (48); therefore, we propose the following fractional-order model for the monkeypox virus transmission model using the Caputo–Fabrizio fractional-order derivative;

(2) $$\begin{array}{l} \{\begin{array}{l} {}^{CF}D_{t}^{\alpha }S_{h}\left(t\right)=\Lambda_{h}+\xi_{h}R_{h}+\theta_{h}Q_{h}-\lambda_{h}S_{h}-\mu_{h}S_{h},\\ {}^{CF}D_{t}^{\alpha }E_{h}\left(t\right)=\lambda_{h}S_{h}-\gamma_{h}E_{h}-\phi_{h}E_{h}-\mu_{h}E_{h},\\ {}^{CF}D_{t}^{\alpha }I_{h}\left(t\right)=\phi_{h}E_{h}-\left(\psi_{h}+\mu_{h}+\nu_{h}\right)I_{h},\\ {}^{CF}D_{t}^{\alpha }Q_{h}\left(t\right)=\gamma_{h}E_{h}-\left(\theta_{h}+\delta_{h}+\mu_{h}+\nu_{h}\right)Q_{h},\\ {}^{CF}D_{t}^{\alpha }R_{h}\left(t\right)=\psi_{h}I_{h}+\delta_{h}Q_{h}-\xi_{h}R_{h}-\mu_{h}R_{h},\\ {}^{CF}D_{t}^{\alpha }S_{r}\left(t\right)=\Lambda_{r}-\lambda_{r}S_{r}-\mu_{r}S_{r}\\ {}^{CF}D_{t}^{\alpha }E_{r}\left(t\right)=\lambda_{r}S_{r}-\phi_{r}E_{r}-\mu_{r}E_{r},\\ {}^{CF}D_{t}^{\alpha }I_{r}\left(t\right)=\phi_{r}E_{r}-\left(\mu_{r}+\nu_{r}\right)I_{r}. \end{array} \end{array}$$

**Figure 1:**
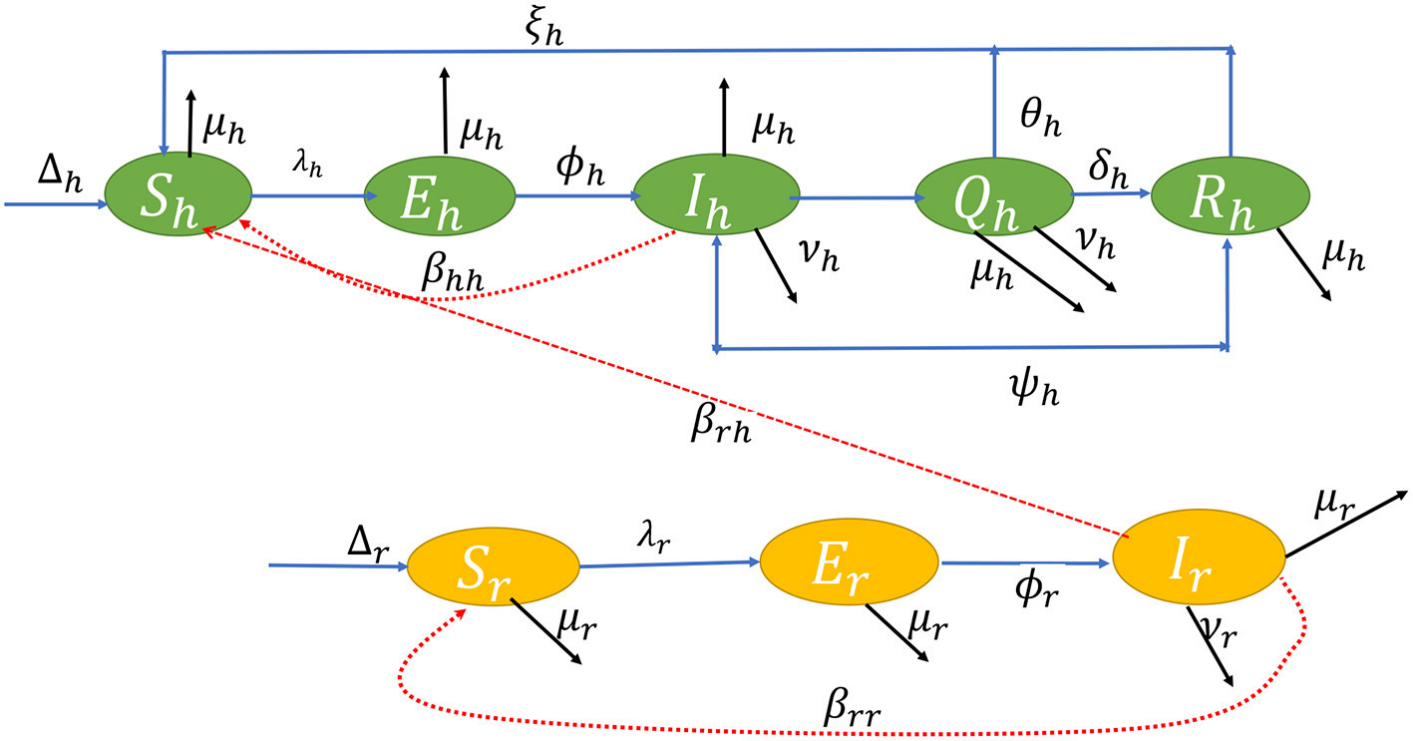
Transfer diagram of the dynamic transmission of the monkeypox virus.

The flow diagram of the model equation is presented in Figure 1 while the parameters used in the model and their signification is presented in Table 1.

**Table 1:** Interpretation of parameters in the model.

**Parameter**	**Interpretation**
Λ_*h*_	Human recruitment rate
Λ_*r*_	Rodent recruitment rate
ξ_*h*_	Immunity loss rate for human
θ_*h*_	Undetected rate of human after diagnosis
μ_*h*_, μ_*r*_	Natural death rate for humans and rodents
ν_*h*_, ν_*r*_	Disease-induced death rate for humans and rodents
ϕ_*h*_, ϕ_*r*_	The rate at which humans and rodents move from exposed to infectious stage
ψ_*h*_	The rate of humans recovery from monkeypox
γ_*h*_	The rate of identifying as suspected case of monkeypox
δ_*h*_	The rate of moving from isolated to recovered class
β_*rh*_	The rate of transmission within rodents and humans
β_*hh*_	The rate of transmission within humans
β_*rr*_	The rate of transmission within rodents

## 4. Dynamics of the model

In this section, we focus on the dynamics of the solutions for the suggested models (1) and (2) that are positive, bounded, and invariant. In an epidemiological model, it is important to evaluate the population survival and the expansion that is naturally constrained by scarce resources. As a result, we demonstrate the following theorem.

**Theorem 1**. The solution of (1) along with initial conditions is positively invariant and bounded in ${\text{R}}_{+}^{8}$. Therefore,

(3) $$\begin{array}{l} \{\begin{array}{l} \lim_{t\to \infty }\sup S_{h}\left(t\right)\leq S_{h\infty }=\frac{\Lambda_{h}+\theta_{h}Q_{h\infty }+\xi_{h}R_{h\infty }}{\lambda_{h}+\mu_{h}},\\ \lim_{t\to \infty }\sup E_{h}\left(t\right)\leq E_{h\infty }=\frac{\lambda_{h}S_{h\infty }}{\left(\xi_{h}+\phi_{h}+\mu_{h}\right)},\\ \lim_{t\to \infty }\sup I_{h}\left(t\right)\leq I_{h\infty }=\frac{\phi_{h}E_{h\infty }}{\left(\psi_{h}+\eta_{h}+\mu_{h}\right)},\\ \lim_{t\to \infty }\sup Q_{h}\left(t\right)\leq Q_{h\infty }=\frac{\gamma_{h}E_{h\infty }}{\left(\theta_{h}+\delta_{h}+\mu_{h}+\nu_{h}\right)},\\ \lim_{t\to \infty }\sup R_{h}\left(t\right)\leq R_{h\infty }=\frac{\psi_{h}I_{h\infty }+\delta_{h}Q_{h\infty }}{\left(\xi_{h}+\mu_{h}\right)},\\ \lim_{t\to \infty }\sup S_{r}\left(t\right)\leq S_{r\infty }=\frac{\Lambda_{r}}{\lambda_{r}+\mu_{r}},\\ \lim_{t\to \infty }\sup E_{r}\left(t\right)\leq E_{r\infty }=\frac{\lambda_{r}S_{r\infty }}{\left(\phi_{r}+\mu_{r}\right)},\\ \lim_{t\to \infty }\sup I_{r}\left(t\right)\leq I_{r\infty }=\frac{\phi_{r}E_{r\infty }}{\left(\nu_{r}+\mu_{r}\right)}. \end{array} \end{array}$$

**Proof**. Using the results in Lin (49) and taking into account the initial values given, from model (2), we obtain

(4) $$\begin{array}{l} \{\begin{array}{l} {}^{CF}D_{t}^{\alpha }S_{h}\left(t\right){|}_{S_{h}\left(0\right)}=\Lambda_{h}+\xi_{h}R_{h}+\theta_{h}Q_{h}\geq 0,\\ {}^{CF}D_{t}^{\alpha }E_{h}\left(t\right){|}_{E_{h}\left(0\right)}=\lambda_{h}S_{h}\geq 0,\\ {}^{CF}D_{t}^{\alpha }I_{h}\left(t\right){|}_{I_{h}\left(0\right)}=\phi_{h}E_{h}\geq 0,\\ {}^{CF}D_{t}^{\alpha }I_{h}\left(t\right){|}_{Q_{h}\left(0\right)}=\gamma_{h}E_{h}\geq 0,\\ {}^{CF}D_{t}^{\alpha }R_{h}\left(t\right){|}_{R_{h}\left(0\right)}=\psi_{h}I_{h}+\delta_{h}Q_{h}\geq 0,\\ {}^{CF}D_{t}^{\alpha }S_{v}\left(t\right){|}_{S_{r}\left(0\right)}=\Lambda_{r}\geq 0,\\ {}^{CF}D_{t}^{\alpha }E_{v}\left(t\right){|}_{E_{r}\left(0\right)}=\lambda_{r}S_{r}\geq 0,\\ {}^{CF}D_{t}^{\alpha }I_{v}\left(t\right){|}_{I_{r}\left(0\right)}=\phi_{r}E_{r}\geq 0. \end{array} \end{array}$$

From Equation (4), we can see that *S*_*h*_(0) > 0, *E*_*h*_(0) > 0, *I*_*h*_(0) > 0, *R*_*h*_(0) > 0, *S*_*v*_(0) > 0, *E*_*v*_(0) > 0, *I*_*v*_(0) > 0, for all *t* > 0. From Equation (2), the first equation gives

$${}^{CF}D_{t}^{\alpha }S_{h}\left(t\right)\leq \Lambda_{h}+\xi_{h}R_{h}+\theta_{h}Q_{h}-\lambda_{h}S_{h}-\mu_{h}S_{h}\geq 0.$$

Then, by applying the fractional comparison technique, we obtain the first estimate of Equation (4). We continue for the second equation of the system of Equation (2), we obtain

$${}^{CF}D_{t}^{\alpha }E_{h}\left(t\right)\leq \lambda_{h}S_{h}-\gamma_{h}E_{h}-\phi_{h}E_{h}-\mu_{h}E_{h}\geq 0.$$

Therefore, we get the second estimate of Equation (1). We continue again for the third equation of the system of Equation (2), we obtain

$${}^{CF}D_{t}^{\alpha }I_{h}\left(t\right)\leq \phi_{h}E_{h}-\left(\psi_{h}+\mu_{h}+\nu_{h}\right)I_{h}\geq 0,$$

and, consequently, we obtain the third estimate of Equation (4). Similarly, for the fourth to eighth equation, we obtain the estimate of Equation (4). Hence, Theorem 1 is complete.

### 4.1. Monkeypox equilibrium state

The monkeypox model is studied by obtaining the equilibrium states. To verify the existence of the equilibrium points, the derivatives of the model on the right-hand side are set to zero, which provides the monkeypox disease free equilibrium points.

We assume *E*_*h*_, *E*_*r*_, *I*_*h*_, *I*_*r*_, *Q*_*h*_, *R*_*h*_, *S*_*h*_, *S*_*r*_ be the solution to the monkeypox model with the initial condition in a feasible region such that

(5) $$\begin{array}{l} \Gamma_{h}=E_{h},I_{h},Q_{h},R_{h},S_{h}\in {\mathbb{R}}^{5}:N_{h}=\frac{\Lambda_{h}}{\mu_{h}}, \end{array}$$

(6) $$\begin{array}{l} \;\Gamma_{r}=E_{r},I_{r},S_{r}\in {\mathbb{R}}^{3}:N_{r}=\frac{\Lambda_{r}}{\mu_{r}}, \end{array}$$

where the human population is represented as

(7) $$\begin{array}{l} N_{h}=E_{h}\left(t\right)+I_{h}\left(t\right)+Q_{h}\left(t\right)+R_{h}\left(t\right)+S_{h}\left(t\right), \end{array}$$

and the rodent population, respectively,

(8) $$\begin{array}{l} N_{r}=E_{r}\left(t\right)+I_{r}\left(t\right)+S_{r}\left(t\right). \end{array}$$

To achieve the disease-free equilibrium state, the derivatives are set to zero as seen in (10) to obtain

(9) $$\begin{array}{l} E^{*}=\left(E_{h}^{*},E_{r}^{*},I_{h}^{*},I_{r}^{*},Q_{h}^{*},R_{h}^{*},S_{h}^{*},S_{r}^{*}\right). \end{array}$$

By setting the derivatives to zero, we obtain

(10) $$\begin{array}{l} \frac{dE_{h}}{dt}=\frac{dE_{r}}{dt}=\frac{dI_{h}}{dt}=\frac{dI_{r}}{dt}=\frac{dQ_{h}}{dt}=\frac{dR_{h}}{dt}=\frac{dS_{h}}{dt}=\frac{dS_{r}}{dt}=0; \end{array}$$

hence, Equation (9) is represented as

(11) $$\begin{array}{l} E^{*}=\left(0,0,0,0,0,0,\frac{\Lambda_{h}}{\mu_{h}},\frac{\Lambda_{r}}{\mu_{r}}\right). \end{array}$$

This equation describes a population free of monkeypox infection and is denoted as E^*^

## 5. The basic reproduction number

We derive the basic reproduction number ℜ_0_ by using the next-generation matrix approach (25). Since *E*_*h*_, *I*_*h*_, *Q*_*h*_, and *I*_*r*_ are the disease-infected classes, hence,

(12) $$\begin{array}{l} f=\left(\begin{array}{c} 0\\ \lambda_{h}S_{h}\\ 0\\ 0\\ 0\\ 0\\ 0\\ 0 \end{array}\right),v=\left(\begin{array}{c} -\Lambda_{h}-\xi_{h}R_{h}-\theta_{h}Q_{h}+\lambda_{h}S_{h}+\mu_{h}S_{h}\\ \gamma_{h}E_{h}+\phi_{h}E_{h}+\mu_{h}E_{h}\\ -\phi_{h}E_{h}+\left(\psi_{h}+\mu_{h}+\nu_{h}\right)I_{h}\\ -\gamma_{h}E_{h}+\left(\theta_{h}+\delta_{h}+\mu_{h}+\nu_{h}\right)Q_{h}\\ -\psi_{h}I_{h}-\delta_{h}Q_{h}+\xi_{h}R_{h}+\mu_{h}R_{h}\\ -\Lambda_{r}+\lambda_{r}S_{r}+\mu_{r}S_{r}\\ -\lambda_{r}S_{r}+\phi_{r}E_{r}+\mu_{r}E_{r}\\ -\phi_{r}E_{r}+\left(\mu_{r}+\nu_{r}\right)I_{r} \end{array}\right). \end{array}$$

(13) $$\begin{array}{ll} F= & \left(\begin{array}{ccccc} 0 & 0 & \frac{\beta_{hh}\Lambda_{h}}{\mu_{h}} & 0 & \frac{\beta_{hh}\Lambda_{h}}{\mu_{h}}\\ 0 & 0 & 0 & 0 & 0\\ 0 & 0 & 0 & 0 & 0\\ 0 & 0 & 0 & 0 & 0 \end{array}\right),\\ V= & \left(\begin{array}{cccc} \gamma_{h}+\phi_{h}+\mu_{h} & 0 & 0 & 0\\ -\phi_{h} & \psi_{h}+\mu_{h}+\nu_{h} & 0 & 0\\ -\gamma_{h} & 0 & \theta_{h}+\delta_{h}+\mu_{h}+\nu_{h} & 0\\ 0 & 0 & 0 & \mu_{r}+\nu_{r} \end{array}\right). \end{array}$$

(14) $$\begin{array}{l} V^{-1}=\left[\begin{array}{cccc} \frac{1}{\gamma_{1}+\mu_{1}+\phi_{1}} & 0 & 0 & 0\\ \frac{\phi_{1}}{\gamma_{1}\mu_{1}+\gamma_{1}\nu_{1}+\gamma_{1}\psi_{1}+\mu^{2}+\nu_{1}\mu_{1}+\mu_{1}\phi_{1}+\mu_{1}\psi_{1}+\nu_{1}\phi_{1}+\psi_{1}\phi_{1}} & \frac{1}{\nu_{1}+\mu_{1}+\psi_{1}} & 0 & 0\\ \frac{\gamma_{1}}{\gamma_{1}\delta_{1}+\delta_{1}\mu_{1}+\delta_{1}\phi_{1}+\gamma_{1}\mu_{1}+\nu_{1}\gamma_{1}+\gamma_{1}\theta_{1}+\mu_{1}^{2}+\mu_{1}\nu_{1}+\mu_{1}\phi_{1}+\mu_{1}\theta_{1}+\nu_{1}\phi_{1}+\theta_{1}\phi_{1}} & 0 & \frac{1}{\nu_{1}+\mu_{1}+\theta_{1}+\delta_{1}} & 0\\ 0 & 0 & 0 & \frac{1}{\nu_{2}+\mu_{2}} \end{array}\right]. \end{array}$$

The next-generation matrix (G) is given by

(15) $$\begin{array}{l} G=F.V^{-1}=\left[\begin{array}{cccc} \frac{\beta_{1}\lambda_{1}\phi_{1}}{N_{h}\mu_{1}\left(\gamma_{1}\mu_{1}+\gamma_{1}\nu_{1}+\gamma_{1}\psi_{1}+\mu^{2}+\nu_{1}\mu_{1}+\mu_{1}\phi_{1}+\mu_{1}\psi_{1}+\nu_{1}\phi_{1}+\psi_{1}\phi_{1}\right)} & \frac{\beta_{1}\lambda_{1}}{N_{h}\mu_{1}\left(\nu_{1}+\mu_{1}+\psi_{1}\right)} & 0 & \frac{\beta_{1}\lambda_{1}}{N_{h}\mu_{1}\left(\nu_{1}+\mu_{1}\right)}\\ 0 & 0 & 0 & 0\\ 0 & 0 & 0 & 0\\ 0 & 0 & 0 & 0 \end{array}\right]. \end{array}$$

The basic reproduction number ${ℜ}_{0}$ is the dominant eigenvalue (spectral radius) of the next-generation matrix G, that is, ℜ_0_ = ρ(*G*)

$${ℜ}_{0}=\frac{\beta_{hh}\Lambda_{h}\phi_{h}}{\mu_{h}\left(\gamma_{h}+\phi_{h}+\mu_{h}\right)\left(\psi_{1}+\mu_{h}+\nu_{h}\right)}$$

### 5.1. Stability of monkeypox-free equilibrium (MFE)

Investigating the stability of the monkeypox disease-free equilibrium, we compute the Jacobian matrix of the system at the disease-free equilibrium by obtaining the eigenvalues, which will be used to determine the stability of the model.

(16) $$\begin{array}{l} J_{E^{*}}=\\ \left(\begin{array}{cccccccc} -\frac{\beta_{hh}I_{h}+\beta_{rh}I_{r}}{N_{h}}-\mu_{h} & 0 & 0 & \theta_{h} & \xi_{h} & 0 & 0 & -\frac{\left(\beta_{hh}+\beta_{rh}\right)S_{h}}{N_{h}}\\ \frac{\beta_{hh}I_{h}+\beta_{rh}I_{r}}{N_{h}} & \zeta_{1} & 0 & 0 & 0 & 0 & 0 & \frac{\left(\beta_{hh}+\beta_{rh}\right)S_{h}}{N_{h}}\\ 0 & \varphi_{h} & 0 & 0 & 0 & 0 & 0 & 0\\ 0 & \gamma_{h} & 0 & \zeta_{2} & 0 & 0 & 0 & 0\\ 0 & 0 & 0 & \delta_{h} & -\mu_{h}-\xi_{h} & 0 & 0 & 0\\ 0 & 0 & 0 & 0 & 0 & -\frac{\beta_{rr}I_{r}}{N_{r}}-\mu_{r} & 0 & -\frac{\beta_{rr}S_{r}}{N_{r}}\\ 0 & 0 & 0 & 0 & 0 & \frac{\beta_{rr}I_{r}}{N_{r}} & -\varphi_{r}-\mu_{r} & \frac{\beta_{rr}S_{r}}{N_{r}}\\ 0 & 0 & 0 & 0 & 0 & 0 & \varphi_{r} & -\mu_{r}-\nu_{r} \end{array}\right), \end{array}$$

where ζ_1_ and ζ_2_ are represented in Equations (17) and (18)

(17) $$\begin{array}{l} \zeta_{1}=-\gamma_{h}-\varphi_{h}-\mu_{h}, \end{array}$$

(18) $$\begin{array}{l} \zeta_{2}=-\theta_{h}-\delta_{h}-\mu_{h}-\nu_{h}. \end{array}$$

Evaluating $J_{E^{*}}$ at the monkeypox-free equilibrium (MFE), we obtain

(19) $$\begin{array}{l} J_{MFE^{*}}\\ =\left(\begin{array}{cccccccc} -\mu_{h} & 0 & 0 & \theta_{h} & \xi_{h} & 0 & 0 & -\frac{\left(\beta_{hh}+\beta_{rh}\right)\Lambda_{h}}{N_{h}\mu_{h}}\\ 0 & -\gamma_{h}-\varphi_{h}-\mu_{h} & 0 & 0 & 0 & 0 & 0 & \frac{\left(\beta_{hh}+\beta_{rh}\right)\Lambda_{h}}{N_{h}\mu_{h}}\\ 0 & \varphi_{h} & 0 & 0 & 0 & 0 & 0 & 0\\ 0 & \gamma_{h} & 0 & -\theta_{h}-\delta_{h}-\mu_{h}-\nu_{h} & 0 & 0 & 0 & 0\\ 0 & 0 & 0 & \delta_{h} & -\mu_{h}-\xi_{h} & 0 & 0 & 0\\ 0 & 0 & 0 & 0 & 0 & -\mu_{r} & 0 & -\frac{\beta_{rr}\Lambda_{r}}{N_{r}\mu_{r}}\\ 0 & 0 & 0 & 0 & 0 & 0 & -\varphi_{r}-\mu_{r} & \frac{\beta_{rr}\Lambda_{r}}{N_{r}\mu_{r}}\\ 0 & 0 & 0 & 0 & 0 & 0 & \varphi_{r} & -\mu_{r}-\nu_{r} \end{array}\right). \end{array}$$

We compute the eigenvalues from the $J_{MFE^{*}}$ using the characteristic polynomial of *O*^8^, which will not be represented as a result of its lengthiness. The eigenvalues and characteristic polynomial are calculated by $\left|J_{MFE^{*}}-I\right|$, where *I* is an 8 × 8 unit matrix, and the values of λ are obtained:

(20) $$\begin{array}{l} \lambda =\left(\begin{array}{c} 0\\ -\mu_{h}\\ -\mu_{h}-\xi_{h}\\ -\mu_{r}\\ -\gamma_{h}-\varphi_{h}-\mu_{h}\\ -\theta_{h}-\delta_{h}-\mu_{h}-\nu_{h}\\ \frac{-2N_{r}\mu_{r}^{2}+\left(-\nu_{r}-\varphi_{r}\right)N_{r}\mu_{r}+\sqrt{\mu_{r}\left(N_{r}{\left(\nu_{r}-\varphi_{r}\right)}^{2}\mu_{r}+4\Lambda_{r}\beta_{rr}\varphi_{r}\right)N_{r}}}{2N_{r}\mu_{r}}\\ \frac{-2N_{r}\mu_{r}^{2}+\left(-\nu_{r}-\varphi_{r}\right)N_{r}\mu_{r}-\sqrt{\mu_{r}\left(N_{r}{\left(\nu_{r}-\varphi_{r}\right)}^{2}\mu_{r}+4\Lambda_{r}\beta_{rr}\varphi_{r}\right)N_{r}}}{2N_{r}\mu_{r}} \end{array}\right) \end{array}$$

Let Δ_1_ *and* Δ_2_ be well represented from Equation (20) in Equations (21) and (22)

(21) $$\begin{array}{l} \Delta_{1}=\mu_{r}\left(N_{r}{\left(\nu_{r}-\varphi_{r}\right)}^{2}\mu_{r}+4\Lambda_{r}\beta_{rr}\varphi_{r}\right)N_{r}, \end{array}$$

(22) $$\begin{array}{l} \Delta_{2}=2N_{r}\mu_{r}^{2}+\left(-\nu_{r}-\varphi_{r}\right)N_{r}\mu_{r}. \end{array}$$

Then,

(23) $$\begin{array}{l} \lambda_{1}=0, \end{array}$$

(24) $$\begin{array}{l} \lambda_{2}=-\mu_{h}, \end{array}$$

(25) $$\begin{array}{l} \lambda_{3}=-\left(\mu_{h}+\xi_{h}\right), \end{array}$$

(26) $$\begin{array}{l} \lambda_{4}=-\mu_{r} \end{array}$$

(27) $$\begin{array}{l} \lambda_{5}=-\left(\gamma_{h}+\varphi_{h}+\mu_{h}\right), \end{array}$$

(28) $$\begin{array}{l} \lambda_{6}=-\left(\theta_{h}+\delta_{h}+\mu_{h}+\nu_{h}\right), \end{array}$$

(29) $$\begin{array}{l} \lambda_{7}=\frac{-\Delta_{2}+\sqrt{\Delta_{1}}}{2N_{r}\mu_{r}}, \end{array}$$

(30) $$\begin{array}{l} \lambda_{8}=\frac{-\Delta_{2}-\sqrt{\Delta_{1}}}{2N_{r}\mu_{r}}. \end{array}$$

From the calculated eigenvalues, we obtain negative real parts, that is, the monkeypox-free equilibrium is asymptotically stable if

(31) $$\begin{array}{l} \frac{-\Delta_{2}-\sqrt{\Delta_{1}}}{2N_{r}\mu_{r}}<0. \end{array}$$

Upon simplification, we obtain Equation (31):

(32) $$\begin{array}{l} \frac{N_{r}\mu_{r}{\left(2\mu_{r}-\nu_{r}-\varphi_{r}\right)}^{2}}{N_{r}\mu_{r}{\left(\nu_{r}-\varphi_{r}\right)}^{2}+4\Lambda_{r}\beta_{rr}\varphi_{r}}<1. \end{array}$$

Therefore, the monkeypox-free equilibrium state is asymptotically stable.

### 5.2. Global stability of the equilibrium state

If ℜ_0_ < 1, then the monkeypox-free equilibrium is globally asymptotically stable; otherwise, it is unstable. This is proven by the Lyapunov function such that

(33) $$\begin{array}{l} L\left(E_{h}\right)=E_{h} \end{array}$$

Differentiating, we obtain

(34) $$\begin{array}{l} L^{\prime }\left(E_{h}\right)=E_{h}^{{}^{\prime }} \end{array}$$

(35) $$\begin{array}{l} \;=\lambda_{h}S_{h}-\gamma_{h}E_{h}-\phi_{h}E_{h}-\mu_{h}E_{h} \end{array}$$

(36) $$\begin{array}{l} \;=\lambda_{h}S_{h}-\left(\gamma_{h}+\phi_{h}+\mu_{h}\right)E_{h}. \end{array}$$

At the disease-free equilibrium state as seen in Equation (11), $S_{h}=\frac{\Lambda_{h}}{\mu_{h}},$

(37) $$\begin{array}{l} L^{\prime }\left(E_{h}\right)=\lambda_{h}\left(\frac{\Lambda_{h}}{\mu_{h}}\right)-\left(\gamma_{h}-\phi_{h}-\mu_{h}\right)E_{h} \end{array}$$

(38) $$\begin{array}{l} E_{h}^{{}^{\prime }}=\left(\gamma_{h}+\phi_{h}+\mu_{h}\right)\left[\frac{\Lambda_{h}\lambda_{h}}{\mu_{h}\left(\gamma_{h}+\phi_{h}+\mu_{h}\right)E_{h}}-1\right]E_{h} \end{array}$$

(39) $$\begin{array}{l} E_{h}^{{}^{\prime }}=\left(\gamma_{h}+\phi_{h}+\mu_{h}\right)\left({ℜ}_{0}-1\right)E_{h}\leq 0\text{ if }{ℜ}_{0}\leq 0. \end{array}$$

From the result obtained in Equation (39), we can see that $E_{h}^{{}^{\prime }}\leq 0$ provided ℜ_0_ ≤ 0 as well as $E_{h}^{{}^{\prime }}=0$ provided that ℜ_0_ = 0 or *E*_*h*_ = 0. Global stability of the disease-free equilibrium is asymptotically stable, if ℜ_0_ ≤ 0; otherwise, it is unstable.

## 6. Endemic equilibrium state

The endemic equilibrium state occurs when the rate of infection persists in the population and it is represented in Equations (40 - 47) by $E_{h}^{**},E_{r}^{**},I_{h}^{**},I_{r}^{**},Q_{h}^{**},R_{h}^{**},S_{h}^{**},S_{r}^{**}.$

(40) $$\begin{array}{l} E_{h}^{**}=\frac{\left[\mu_{h}^{3}+k_{1}\mu_{h}^{2}+\left(k_{2}+k_{3}\right)\mu_{h}+k_{4}\right]\Lambda_{h}\lambda_{h}}{\mu_{h}^{5}+p_{1}\mu_{h}^{4}+p_{2}\cdot \mu_{h}^{3}+p_{3}\cdot \mu_{h}^{2}+\mu_{h}\cdot p_{4}+\lambda_{h}\nu_{h}\xi_{h}\cdot p_{5}} \end{array}$$

(41) $$\begin{array}{l} E_{r}^{**}=\frac{\lambda_{r}\Lambda_{r}}{\left(\mu_{r}+\varphi_{r}\right)\left(\mu_{r}+\lambda_{r}\right)} \end{array}$$

(42) $$\begin{array}{l} I_{h}^{**}=\frac{\left(\mu_{h}^{2}+\left(\delta_{h}+\nu_{h}+\theta_{h}+\xi_{h}\right)\mu_{h}+\delta_{h}\xi_{h}+\nu_{h}\xi_{h}+\theta_{h}\xi_{h}\right)\Lambda_{h}\lambda_{h}\varphi_{h}}{\mu_{h}^{5}+p_{1}\mu_{h}^{4}+p_{2}\cdot \mu_{h}^{3}+p_{3}\cdot \mu_{h}^{2}+\mu_{h}\cdot p_{4}+\lambda_{h}\nu_{h}\xi_{h}\cdot p_{5}+p_{6}} \end{array}$$

(43) $$\begin{array}{l} I_{r}^{**}=\frac{\varphi_{r}\lambda_{r}\Lambda_{r}}{\lambda_{r}\mu_{r}^{2}+\lambda_{r}\mu_{r}\nu_{r}+\lambda_{r}\mu_{r}\varphi_{r}+\lambda_{r}\nu_{r}\varphi_{r}+\mu_{r}^{3}+\mu_{r}^{2}\nu_{r}+\mu_{r}^{2}\varphi_{r}+\mu_{r}\nu_{r}\varphi_{r}} \end{array}$$

(44) $$\begin{array}{l} Q_{h}^{**}=\frac{\left(\gamma_{h}\mu_{h}^{2}+\gamma_{h}\left(\nu_{h}+\psi_{h}+\xi_{h}\right)\mu_{h}+\gamma_{h}\left(\nu_{h}\xi_{h}+\psi_{h}\xi_{h}\right)\right)\Lambda_{h}\lambda_{h}}{\mu_{h}^{5}+p_{1}\mu_{h}^{4}+p_{2}\cdot \mu_{h}^{3}+p_{3}\cdot \mu_{h}^{2}+\mu_{h}\cdot p_{4}+\lambda_{h}\nu_{h}\xi_{h}\cdot p_{5}+p_{6}} \end{array}$$

(45) $$\begin{array}{l} R_{h}^{**}=\\ \frac{\left(\delta_{h}\psi_{h}+\mu_{h}\psi_{h}+\nu_{h}\psi_{h}+\psi_{h}\theta_{h}\right)\lambda_{h}\Lambda_{h}\varphi_{h}+\left(\delta_{h}\gamma_{h}\mu_{h}+\delta_{h}\gamma_{h}\nu_{h}+\delta_{h}\gamma_{h}\psi_{h}\right)\lambda_{h}\Lambda_{h}}{\mu_{h}^{5}+p_{1}\mu_{h}^{4}+p_{2}\cdot \mu_{h}^{3}+p_{3}\cdot \mu_{h}^{2}+\mu_{h}\cdot p_{4}+\lambda_{h}\nu_{h}\xi_{h}\cdot p_{5}+p_{6}} \end{array}$$

(46) $$\begin{array}{l} S_{h}^{**}=\frac{\Lambda_{h}\mu_{h}^{4}+\Lambda_{h}\cdot h_{1}\cdot \mu_{h}^{3}+\Lambda_{h}\cdot h_{2}\cdot \mu_{h}^{2}+\Lambda_{h}\cdot h_{3}\cdot \mu_{h}+\Lambda_{h}\cdot h_{4}}{\mu_{h}^{5}+p_{1}\mu_{h}^{4}+p_{2}\cdot \mu_{h}^{3}+p_{3}\cdot \mu_{h}^{2}+\mu_{h}\cdot p_{4}+\lambda_{h}\nu_{h}\xi_{h}\cdot p_{5}+p_{6}} \end{array}$$

(47) $$\begin{array}{l} S_{r}^{**}=\frac{\Lambda_{r}}{\lambda_{r}+\mu_{r}}, \end{array}$$

where

(48) $$\begin{array}{l} d_{1}=\left(\psi_{h}+\nu_{h}\right),\\ d_{2}=\left(\delta_{h}+\nu_{h}+\theta_{h}+\xi_{h}\right),\\ d_{3}=\left(\delta_{h}+\nu_{h}+\theta_{h}\right),\\ k_{1}=\left(\psi_{h}+2\nu_{h}+\delta_{h}+\theta_{h}+\xi_{h}\right)\\ k_{2}=d_{1}+d_{2},\\ k_{3}=\xi_{h}\cdot d_{3},\\ k_{4}=d_{1}\cdot \xi_{h}\cdot d_{3},\\ p_{1}=\delta_{h}+\gamma_{h}+\lambda_{h}+2\nu_{h}+\psi_{h}+\theta_{h}+\varphi_{h}+\xi_{h},\\ p_{2}=\delta_{h}\gamma_{h}+\delta_{h}\lambda_{h}+\delta_{h}\nu_{h}+\delta_{h}\psi_{h}+\delta_{h}\varphi_{h}+\delta_{h}\xi_{h}+\gamma_{h}\lambda_{h}\\ \;+2\gamma_{h}\nu_{h}+\gamma_{h}\psi_{h}+\gamma_{h}\theta_{h}+\gamma_{h}\xi_{h}+2\lambda_{h}\nu_{h}+\lambda_{h}\psi_{h}+\lambda_{h}\theta_{h}\\ \;+\lambda_{h}\varphi_{h}+\lambda_{h}\xi_{h}+\nu_{h}^{2}+\nu_{h}\psi_{h}+\nu_{h}\theta_{h}+2\nu_{h}\varphi_{h}+2\nu_{h}\xi_{h}\\ \;+\psi_{h}\theta_{h}+\psi_{h}\varphi_{h}+\psi_{h}\xi_{h}\\ \;+\theta_{h}\varphi_{h}+\theta_{h}\xi_{h}+\varphi_{h}\xi_{h},\\ p_{3}=\delta_{h}\gamma_{h}\lambda_{h}+\delta_{h}\gamma_{h}\nu_{h}+\delta_{h}\gamma_{h}\psi_{h}+\delta_{h}\gamma_{h}\xi_{h}+\delta_{h}\lambda_{h}\nu_{h}+\delta_{h}\lambda_{h}\psi_{h}\\ \;+\delta_{h}\lambda_{h}\varphi_{h}+\delta_{h}\lambda_{h}\xi_{h}+\delta_{h}\nu_{h}\varphi_{h}+\delta_{h}\nu_{h}\xi_{h}+\delta_{h}\psi_{h}\varphi_{h}+\delta_{h}\psi_{h}\xi_{h}\\ \;+\delta_{h}\varphi_{h}\xi_{h}+2\gamma_{h}\lambda_{h}\nu_{h}+\gamma_{h}\lambda_{h}\psi_{h}+\gamma_{h}\lambda_{h}\xi_{h}+\gamma_{h}\nu_{h}^{2}+\gamma_{h}\nu_{h}\psi_{h}\\ \;+\gamma_{h}\nu_{h}\theta_{h}+2\gamma_{h}\nu_{h}\xi_{h}+\gamma_{h}\psi_{h}\theta_{h}+\gamma_{h}\psi_{h}\xi_{h}+\gamma_{h}\theta_{h}\xi_{h}+\lambda_{h}\nu_{h}^{2}\\ \;+\lambda_{h}\nu_{h}\psi_{h}+\lambda_{h}\nu_{h}\theta_{h}+2\lambda_{h}\nu_{h}\varphi_{h}+2\lambda_{h}\nu_{h}\xi_{h}+\lambda_{h}\psi_{h}\theta_{h}+\lambda_{h}\psi_{h}\varphi_{h}\\ \;+\lambda_{h}\psi_{h}\xi_{h}+\lambda_{h}\theta_{h}\varphi_{h}+\lambda_{h}\theta_{h}\xi_{h}+\lambda_{h}\varphi_{h}\xi_{h}+\nu_{h}^{2}\varphi_{h}+\nu_{h}^{2}\xi_{h}+\nu_{h}\psi_{h}\varphi_{h}\\ \;+\nu_{h}\psi_{h}\xi_{h}+\nu_{h}\theta_{h}\varphi_{h}+\nu_{h}\theta_{h}\xi_{h}+2\nu_{h}\varphi_{h}\xi_{h}+\psi_{h}\theta_{h}\varphi_{h}+\psi_{h}\theta_{h}\xi_{h}\\ \;+\psi_{h}\varphi_{h}\xi_{h}+\theta_{h}\varphi_{h}\xi_{h},\\ p_{4}=\delta_{h}\gamma_{h}\lambda_{h}\nu_{h}+\delta_{h}\gamma_{h}\lambda_{h}\psi_{h}+\delta_{h}\gamma_{h}\nu_{h}\xi_{h}+\delta_{h}\gamma_{h}\psi_{h}\xi_{h}+\delta_{h}\lambda_{h}\nu_{h}\varphi_{h}\\ \;+\delta_{h}\lambda_{h}\nu_{h}\xi_{h}+\delta_{h}\lambda_{h}\psi_{h}\varphi_{h}+\delta_{h}\lambda_{h}\psi_{h}\xi_{h}+\delta_{h}\lambda_{h}\varphi_{h}\xi_{h}+\delta_{h}\nu_{h}\varphi_{h}\xi_{h}\\ \;+\delta_{h}\psi_{h}\varphi_{h}\xi_{h}+\gamma_{h}\lambda_{h}\nu_{h}^{2}+\gamma_{h}\lambda_{h}\nu_{h}\psi_{h}+2\gamma_{h}\lambda_{h}\nu_{h}\xi_{h}+\gamma_{h}\lambda_{h}\psi_{h}\xi_{h}\\ \;+\gamma_{h}\nu_{h}^{2}\xi_{h}+\gamma_{h}\nu_{h}\psi_{h}\xi_{h}+\gamma_{h}\nu_{h}\theta_{h}\xi_{h}+\gamma_{h}\psi_{h}\theta_{h}\xi_{h}+\lambda_{h}\nu_{h}^{2}\varphi_{h}+\lambda_{h}\nu_{h}^{2}\xi_{h}\\ \;+\lambda_{h}\nu_{h}\psi_{h}\varphi_{h}+\lambda_{h}\nu_{h}\psi_{h}\xi_{h}+\lambda_{h}\nu_{h}\theta_{h}\varphi_{h}+\lambda_{h}\nu_{h}\theta_{h}\xi_{h}+2\lambda_{h}\nu_{h}\varphi_{h}\xi_{h}\\ \;+\lambda_{h}\psi_{h}\theta_{h}\varphi_{h}+\lambda_{h}\psi_{h}\theta_{h}\xi_{h}+\lambda_{h}\theta_{h}\varphi_{h}\xi_{h}+\nu_{h}^{2}\varphi_{h}\xi_{h}\\ \;+\nu_{h}\psi_{h}\varphi_{h}\xi_{h}+\nu_{h}\theta_{h}\varphi_{h}\xi_{h}+\psi_{h}\theta_{h}\varphi_{h}\xi_{h},\\ p_{5}=\delta_{h}\varphi_{h}+\gamma_{h}\nu_{h}+\gamma_{h}\psi_{h}+\nu_{h}\varphi_{h}+\theta_{h}\varphi_{h},\\ p_{6}=\delta_{h}\lambda_{h}\nu_{h}\varphi_{h}\xi_{h}+\gamma_{h}\lambda_{h}\nu_{h}^{2}\xi_{h}+\gamma_{h}\lambda_{h}\nu_{h}\psi_{h}\xi_{h}+\lambda_{h}\nu_{h}^{2}\varphi_{h}\xi_{h}\\ \;+\lambda_{h}\nu_{h}\theta_{h}\varphi_{h}\xi_{h},\\ h_{1}=\delta_{h}+\gamma_{h}+2\nu_{h}+\psi_{h}+\theta_{h}+\varphi_{h}+\xi_{h},\\ h_{2}=\delta_{h}\gamma_{h}+\delta_{h}\nu_{h}+\delta_{h}\psi_{h}+\delta_{h}\varphi_{h}+\delta_{h}\xi_{h}+2\gamma_{h}\nu_{h}+\gamma_{h}\psi_{h}+\gamma_{h}\theta_{h}\\ \;+\gamma_{h}\xi_{h}+\nu_{h}^{2}+\nu_{h}\psi_{h}+\nu_{h}\theta_{h}+2\nu_{h}\varphi_{h}+2\nu_{h}\xi_{h}+\psi_{h}\theta_{h}+\psi_{h}\varphi_{h}\\ \;+\psi_{h}\xi_{h}+\theta_{h}\varphi_{h}+\theta_{h}\xi_{h}+\varphi_{h}\xi_{h},\\ h_{3}=\delta_{h}\gamma_{h}\nu_{h}+\delta_{h}\gamma_{h}\psi_{h}+\delta_{h}\gamma_{h}\xi_{h}+\delta_{h}\nu_{h}\varphi_{h}+\delta_{h}\nu_{h}\xi_{h}+\delta_{h}\psi_{h}\varphi_{h}\\ \;+\delta_{h}\psi_{h}\xi_{h}+\delta_{h}\varphi_{h}\xi_{h}+\gamma_{h}\nu_{h}^{2}+\gamma_{h}\nu_{h}\psi_{h}+\gamma_{h}\nu_{h}\theta_{h}\\ \;+2\gamma_{h}\nu_{h}\xi_{h}+\gamma_{h}\psi_{h}\theta_{h}+\gamma_{h}\psi_{h}\xi_{h}+\gamma_{h}\theta_{h}\xi_{h}+\nu_{h}^{2}\varphi_{h}\\ \;+\nu_{h}^{2}\xi_{h}+\nu_{h}\psi_{h}\varphi_{h}+\nu_{h}\psi_{h}\xi_{h}+\nu_{h}\theta_{h}\varphi_{h}+\nu_{h}\theta_{h}\xi_{h}+2\nu_{h}\varphi_{h}\xi_{h}\\ \;+\psi_{h}\theta_{h}\varphi_{h}+\psi_{h}\theta_{h}\xi_{h}+\psi_{h}\varphi_{h}\xi_{h}+\theta_{h}\varphi_{h}\xi_{h},\\ h_{4}=\delta_{h}\gamma_{h}\nu_{h}\xi_{h}+\delta_{h}\gamma_{h}\psi_{h}\xi_{h}+\delta_{h}\nu_{h}\varphi_{h}\xi_{h}+\delta_{h}\psi_{h}\varphi_{h}\xi_{h}+\gamma_{h}\nu_{h}^{2}\xi_{h}\\ \;+\gamma_{h}\nu_{h}\psi_{h}\xi_{h}+\gamma_{h}\nu_{h}\theta_{h}\xi_{h}+\gamma_{h}\psi_{h}\theta_{h}\xi_{h}+\nu_{h}^{2}\varphi_{h}\xi_{h}+\nu_{h}\psi_{h}\varphi_{h}\xi_{h}\\ \;+\nu_{h}\theta_{h}\varphi_{h}\xi_{h}+\psi_{h}\theta_{h}\varphi_{h}\xi_{h}, \end{array}$$

## 7. Existence and uniqueness results for the monkeypox transmission model with non-pharmaceutical intervention

We reformulate Equation (2) as follows:

$$\begin{array}{l} \{\begin{array}{c} \Phi_{1}\left(t,S_{h}\left(t\right),E_{h}\left(t\right),I_{h}\left(t\right),Q_{h}\left(t\right),R_{h}\left(t\right),S_{r}\left(t\right),E_{r}\left(t\right),I_{r}\left(t\right)\right)=\Lambda_{h}+\xi_{h}R_{h}\\ +\theta_{h}Q_{h}-\lambda_{h}S_{h}-\mu_{h}S_{h},\\ \Phi_{2}\left(t,S_{h}\left(t\right),E_{h}\left(t\right),I_{h}\left(t\right),Q_{h}\left(t\right),R_{h}\left(t\right),S_{r}\left(t\right),E_{r}\left(t\right),I_{r}\left(t\right)\right)=\lambda_{h}S_{h}\\ -\gamma_{h}E_{h}-\phi_{h}E_{h}-\mu_{h}E_{h},\\ \Phi_{3}\left(t,S_{h}\left(t\right),E_{h}\left(t\right),I_{h}\left(t\right),Q_{h}\left(t\right),R_{h}\left(t\right),S_{r}\left(t\right),E_{r}\left(t\right),I_{r}\left(t\right)\right)=\phi_{h}E_{h}\\ -\left(\psi_{h}+\mu_{h}+\nu_{h}\right)I_{h},\\ \Phi_{4}\left(t,S_{h}\left(t\right),E_{h}\left(t\right),I_{h}\left(t\right),Q_{h}\left(t\right),R_{h}\left(t\right),S_{r}\left(t\right),E_{r}\left(t\right),I_{r}\left(t\right)\right)=\gamma_{h}E_{h}\\ -\left(\theta_{h}+\delta_{h}+\mu_{h}+\nu_{h}\right)Q_{h}\\ \Phi_{5}\left(t,S_{h}\left(t\right),E_{h}\left(t\right),I_{h}\left(t\right),Q_{h}\left(t\right),R_{h}\left(t\right),S_{r}\left(t\right),E_{r}\left(t\right),I_{r}\left(t\right)\right)=\psi_{h}I_{h}\\ +\delta_{h}Q_{h}-\xi_{h}R_{h}-\mu_{h}R_{h},\\ \Phi_{6}\left(t,S_{h}\left(t\right),E_{h}\left(t\right),I_{h}\left(t\right),Q_{h}\left(t\right),R_{h}\left(t\right),S_{r}\left(t\right),E_{r}\left(t\right),I_{r}\left(t\right)\right)=\Lambda_{r}-\lambda_{r}S_{r}\\ -\mu_{r}S_{r}\\ \Phi_{7}\left(t,S_{h}\left(t\right),E_{h}\left(t\right),I_{h}\left(t\right),Q_{h}\left(t\right),R_{h}\left(t\right),S_{r}\left(t\right),E_{r}\left(t\right),I_{r}\left(t\right)\right)=\lambda_{r}S_{r}-\phi_{r}E_{r}\\ -\mu_{r}E_{r},\\ \Phi_{8}\left(t,S_{h}\left(t\right),E_{h}\left(t\right),I_{h}\left(t\right),Q_{h}\left(t\right),R_{h}\left(t\right),S_{r}\left(t\right),E_{r}\left(t\right),I_{r}\left(t\right)\right)=\phi_{r}E_{r}\\ -\left(\mu_{r}+\nu_{r}\right)I_{r}. \end{array} \end{array}$$

From Equation (10), the developed model of Equation (1) can be written in the form

(49) $$\begin{array}{l} \{\begin{array}{l} {}^{CF}D_{t}^{\alpha }\Phi \left(t\right)=ϒ\left(t,\Phi \left(t\right)\right),t\in \left[0,\eta \right],\;0<\alpha \leq 1,\\ \Phi \left(0\right)=\Phi_{0}, \end{array} \end{array}$$

(50) $$\begin{array}{l} \Phi \left(t\right)=\{\begin{array}{l} S_{h}\left(t\right),\\ E_{h}\left(t\right),\\ I_{h}\left(t\right),\\ Q_{h}\left(t\right),\\ R_{h}\left(t\right),\\ S_{r}\left(t\right),\\ E_{r}\left(t\right),\\ I_{r}\left(t\right), \end{array}\Phi_{0}=\{\begin{array}{l} S_{h}\left(0\right),\\ E_{h}\left(0\right),\\ I_{h}\left(0\right),\\ Q_{h}\left(0\right),\\ R_{h}\left(0\right),\\ S_{r}\left(0\right),\\ E_{r}\left(0\right),\\ I_{r}\left(0\right), \end{array} \end{array}$$

therefore,

(51) $$\begin{array}{l} ϒ\left(t,\Phi \left(t\right)\right)=\{\begin{array}{l} \Phi_{1}\left(t,S_{h}\left(t\right),E_{h}\left(t\right),I_{h}\left(t\right),Q_{h}\left(t\right),R_{h}\left(t\right),S_{r}\left(t\right),E_{r}\left(t\right),I_{r}\left(t\right)\right),\\ \Phi_{2}\left(t,S_{h}\left(t\right),E_{h}\left(t\right),I_{h}\left(t\right),Q_{h}\left(t\right),R_{h}\left(t\right),S_{r}\left(t\right),E_{r}\left(t\right),I_{r}\left(t\right)\right),\\ \Phi_{3}\left(t,S_{h}\left(t\right),E_{h}\left(t\right),I_{h}\left(t\right),Q_{h}\left(t\right),R_{h}\left(t\right),S_{r}\left(t\right),E_{r}\left(t\right),I_{r}\left(t\right)\right),\\ \Phi_{4}\left(t,S_{h}\left(t\right),E_{h}\left(t\right),I_{h}\left(t\right),Q_{h}\left(t\right),R_{h}\left(t\right),S_{r}\left(t\right),E_{r}\left(t\right),I_{r}\left(t\right)\right),\\ \Phi_{5}\left(t,S_{h}\left(t\right),E_{h}\left(t\right),I_{h}\left(t\right),Q_{h}\left(t\right),R_{h}\left(t\right),S_{r}\left(t\right),E_{r}\left(t\right),I_{r}\left(t\right)\right),\\ \Phi_{6}\left(t,S_{h}\left(t\right),E_{h}\left(t\right),I_{h}\left(t\right),Q_{h}\left(t\right),R_{h}\left(t\right),S_{r}\left(t\right),E_{r}\left(t\right),I_{r}\left(t\right)\right),\\ \Phi_{7}\left(t,S_{h}\left(t\right),E_{h}\left(t\right),I_{h}\left(t\right),Q_{h}\left(t\right),R_{h}\left(t\right),S_{r}\left(t\right),E_{r}\left(t\right),I_{r}\left(t\right)\right),\\ \Phi_{8}\left(t,S_{h}\left(t\right),E_{h}\left(t\right),I_{h}\left(t\right),Q_{h}\left(t\right),R_{h}\left(t\right),S_{r}\left(t\right),E_{r}\left(t\right),I_{r}\left(t\right)\right). \end{array} \end{array}$$

With the help of Lemma 2.4, Equation (49) yields

(52) $$\begin{array}{l} \{\begin{array}{c} \Phi \left(t\right)=\Phi_{0}\left(t\right)+\frac{2\left(1-\alpha \right)}{2\left(1-\alpha \right)G\left(\alpha \right)}ϒ\left(t,\Phi \left(t\right)\right)+\frac{2\alpha }{\left(2-\alpha \right)G\left(\alpha \right)}\\ \;\times \int_{0}^{t}ϒ\left(s,\Phi \left(s\right)\right)ds. \end{array} \end{array}$$

Furthermore, let ussay ***E*** = *C*([0, η]) is the Banach space, and supposing that the following assumptions hold;

(*H*_1_), there exists a non-negative constant *Q, W*, and *k* ∈ [0, 1) such that

$$ϒ\left(t,\Phi \left(t\right)\right)\leq Q|\Phi {|}^{k}+W.$$

(*H*_2_) There exists a nonnegative constant **C**_ρ_ > 0 for all $\Phi ,\tilde{\Phi }\in E$, then

$$|ϒ\left(t,\Phi \left(t\right)\right)-ϒ\left(t,\tilde{\Phi }\left(t\right)\right)|\leq {\text{C}}_{\rho }\left[\left|\Phi -\tilde{\Phi }\right|\right].$$

Furthermore, let us define operator **A**_*m*_ : ***E*** → ***E*** such that

$${\text{A}}_{m}ℵ\left(t\right)=M_{1}\Phi \left(t\right)+M_{2}\Phi \left(t\right),$$

therefore, we can see that

(53) $$\begin{array}{l} \{\begin{array}{l} M_{1}\Phi \left(t\right)=\Phi_{0}\left(t\right)+\frac{2\left(1-\alpha \right)}{2\left(1-\alpha \right)G\left(\alpha \right)}ϒ\left(t,\Phi \left(t\right)\right),\\ M_{2}\Phi \left(t\right)=\frac{2\alpha }{\left(2-\alpha \right)G\left(\alpha \right)}\int_{0}^{t}ϒ\left(s,\Phi \left(s\right)\right)ds. \end{array} \end{array}$$

From this knowledge, Equation (52) can be written as

(54) $$\begin{array}{l} \{\begin{array}{l} {\text{A}}_{m}\Phi \left(t\right)=\Phi_{0}\left(t\right)+\frac{2\left(1-\alpha \right)}{2\left(1-\alpha \right)G\left(\alpha \right)}ϒ\left(t,\Phi \left(t\right)\right)+\frac{2\alpha }{\left(2-\alpha \right)G\left(\alpha \right)}\\ \;\times \int_{0}^{t}ϒ\left(s,\Phi \left(s\right)\right)ds. \end{array} \end{array}$$

**Theorem 2**. Suppose that (*H*_1_) and (*H*_2_) hold, such that $\frac{2\left(1-\alpha \right)}{2\left(1-\alpha \right)G\left(\alpha \right)}{\text{C}}_{\rho }<1$, then, the monkeypox transmission model with non-pharmaceutical intervention has at least one solution.

**Proof**. For simplicity, we divide the proof into two steps.

Step 1. We prove that operator *M*_1_ is contraction. Then, let $\tilde{\Phi }\in \Omega$, where Ω = {Φ ∈ **Z** : ||Φ|| ≤ ϑ, ϑ > 0} is a close convex set, thus

(55) $$\begin{array}{l} \begin{array}{l} |M_{1}\Phi \left(t\right)-M_{2}\Phi |=\frac{2\left(1-\alpha \right)}{2\left(1-\alpha \right)G\left(\alpha \right)}\max_{\alpha \in \left[0,\eta \right]}\\ \;|ϒ\left(t,\Phi \left(t\right)\right)-ϒ\left(t,\tilde{\Phi }\left(t\right)\right)|,\\ \;\leq \frac{2\left(1-\alpha \right)}{2\left(1-\alpha \right)G\left(\alpha \right)}{\text{C}}_{\rho }||\Phi -\tilde{\Phi }||. \end{array} \end{array}$$

Thus,

$$||M_{1}\Phi -M_{2}\Phi \left(t\right)||\leq \frac{2\left(1-\alpha \right)}{2\left(1-\alpha \right)G\left(\alpha \right)}{\text{C}}_{\rho }||\Phi -\tilde{\Phi }||.$$

Hence, *M*_1_ is contraction since $\frac{2\left(1-\alpha \right)}{2\left(1-\alpha \right)G\left(\alpha \right)}{\text{C}}_{\rho }<1$.

Step 2. We also prove that *M*_2_ is compact and also continuous; for all Φ ∈ Ω, then *M*_2_ will be continuous as Φ is continuous, thus

(56) $$\begin{array}{l} \begin{array}{l} ||M_{2}\left(\Phi \right)||=\max_{t\in \left[0,\eta \right]}|\frac{2\alpha }{\left(2-\alpha \right)G\left(\alpha \right)}\int_{0}^{t}ϒ\left(s,\Phi \left(s\right)\right)ds|,\\ \;\leq \frac{2\alpha }{\left(2-\alpha \right)G\left(\alpha \right)}\eta \int_{0}^{t}|ϒ\left(s,\Phi \left(s\right)\right)|ds.\\ \;\leq \frac{2\alpha }{\left(2-\alpha \right)G\left(\alpha \right)}\eta \left[Q|\Phi {|}^{k}+W\right]. \end{array} \end{array}$$

Hence, *M*_2_ is boundedness. For equicontinuous, let *t*_1_, *t*_2_ ∈ [0, η] such that

(57) $$\begin{array}{l} \begin{array}{l} |\left(M_{2}\Phi \right)\left(t_{1}\right)-\left(M_{2}\Phi \right)\left(t_{2}\right)|=\frac{2\alpha }{\left(2-\alpha \right)G\left(\alpha \right)}\max_{t\in \left[0,\eta \right]}|\int_{0}^{t_{1}}ϒ\left(s,\Phi \left(s\right)\right)ds\\ \;-\int_{0}^{t_{2}}\Phi \left(s,ℵ\left(s\right)\right)ds|\\ \;\leq \frac{2\alpha }{\left(2-\alpha \right)G\left(\alpha \right)}\left[Q|\Phi {|}^{k}+W\right]|t_{1}-t_{2}|. \end{array} \end{array}$$

As *t*_1_ → *t*_2_, then |(*M*_2_Φ)(*t*_1_) − (*M*_2_Φ)(*t*_2_)| → 0, which makes operator *M*_2_ equicontinuous and compact by the Arzela–Ascoli theorem. Therefore, by Lemma 2.3, the existence for the monkeypox transmission model with non-pharmaceutical intervention has at least one solution.   □

**Theorem 3**. Suppose that ∃ is a nonnegative integer Λ_ρ_ is > 0 such that

(58) $$\begin{array}{l} \begin{array}{c} \Lambda_{\rho }=\left[\frac{2\left(1-\alpha \right)}{2\left(1-\alpha \right)G\left(\alpha \right)}{\text{L}}_{\rho }+\frac{2\alpha }{\left(2-\alpha \right)G\left(\alpha \right)}\eta {\text{L}}_{\rho }\right]<1, \end{array} \end{array}$$

then operator **A**_*m*_ has a unique fixed point.

**Proof**. Let $\Phi ,\tilde{\Phi }\in \Omega$, then we say

(59) $$\begin{array}{l} \begin{array}{l} ||{\text{A}}_{m}\Phi -{\text{A}}_{m}\tilde{\Phi }||\leq ||M_{1}\Phi -M_{1}\tilde{\Phi }||+||M_{2}\Phi -M_{2}\tilde{\Phi }||,\\ \leq \frac{2\left(1-\alpha \right)}{2\left(1-\alpha \right)G\left(\alpha \right)}\max_{t\in \left[0,\eta \right]}|ϒ\left(t,\Phi \left(t\right)\right)-ϒ\left(t,\tilde{\Phi }\left(t\right)\right)|\\ +\frac{2\alpha }{\left(2-\alpha \right)G\left(\alpha \right)}\max_{t\in \left[0,\eta \right]}|\int_{0}^{t}ϒ\left(s,\Phi \left(s\right)\right)ds\\ -\int_{0}^{t}ϒ\left(s,\tilde{\Phi }\left(s\right)\right)ds|\\ \leq \left[\frac{2\left(1-\alpha \right)}{2\left(1-\alpha \right)G\left(\alpha \right)}{\text{C}}_{\rho }+\frac{2\alpha }{\left(2-\alpha \right)G\left(\alpha \right)}\eta {\text{C}}_{\rho }\right]||\Phi -\tilde{\Phi }||,\\ =\Lambda_{\rho }||\Phi -\tilde{\Phi }||. \end{array} \end{array}$$

Hence, by the Banach contraction principle, **A**_*m*_ has a unique fixed point. Consequently, the monkeypox transmission model with non-pharmaceutical intervention has a unique solution. □

## 8. Fitting of model to data

We used the available public database to collect our data while the formulated model of Equation (1) includes 16 parameters. To treat the waggliness of the reported daily new cases, we smoothed the data to remove noise from the data set so as to make it suitable for our analysis. The total population of the United Kingdom is 68,530,739 (1), which was used for calculating the initial number of susceptible humans, while the initial value for the number of infected humans was calculated from the reported daily new cases. Other initial values were assumed.

**Figure 2:**
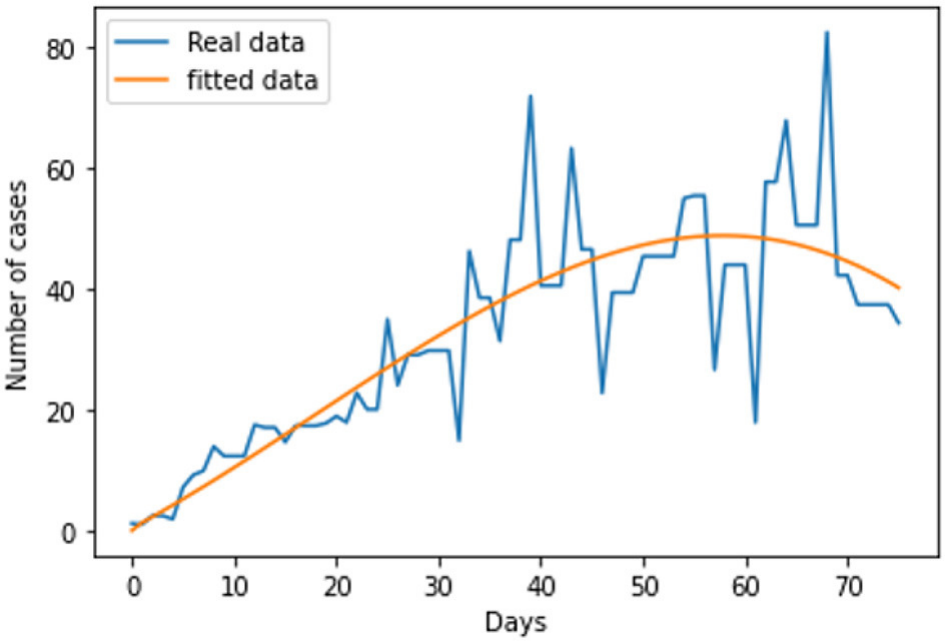
Model fitting.

The link to the data used for this research and the initial values; *S*_*h*_(*t*) = 68530739; *E*_*h*_(*t*) = 0; *I*_*h*_(*t*) = 31412; *Q*_*h*_(*t*) = 0; *S*_*r*_(*t*) = 1074103; *E*_*r*_(*t*) = 1074103; and *I*_*r*_(*t*) = 1074103; can be found in the Data Availability section. The parameters are fitted based on the smoothed reported daily new cases of infected humans from May to August 2022. This information was taken from the United Kingdom public health database (1). The nonlinear least square technique was used to fit the model using python programming. Table 2 shows all of the parameter values that were fitted, and Figure 2 shows the data fitting of the observed smoothed daily new cases.

**Table 2:** Parameter values in the model.

**Parameter**	**Value**	**Source**
Λ_*h*_	8644	Estimated
Λ_*r*_	0.9	Assumed
ξ_*h*_	0.00001	Fitted
θ_*h*_	0.029	Fitted
μ_*r*_	0.00200	(46)
μ_*h*_	0.05	(1)
ν_*h*_, ν_*r*_	0.00008, 0.0001	Fitted
ϕ_*h*_, ϕ_*r*_	0.007	Fitted
ψ_*h*_	0.056	Fitted
γ_*h*_	0.0081	Fitted
δ_*h*_	0.012	Fitted
β_*rh*_	0.000009	Fitted
β_*hh*_	0.00008	Fitted
β_*rr*_	0.0057	Fitted

## 9. Numerical scheme

In this section, we present the numerical results for the monkeypox transmission model with non-pharmaceutical intervention based on the Lagrange interpolation. Details about the numerical scheme is presented in Atangana and Owolabi (50). The Cauchy problem of the CF fractional derivative can be given as:

(60) $$\begin{array}{l} \begin{array}{c} {}^{CF}D_{t}^{\alpha }\Phi \left(t\right)=ϒ\left(t,\Phi \left(t\right)\right), \end{array} \end{array}$$

On the other hand, we can express Equation (60) as

(61) $$\begin{array}{l} \Phi \left(t\right)=\Phi_{0}\left(t\right)+\frac{\left(1-\alpha \right)}{G\left(\alpha \right)}ϒ\left(t,\Phi \left(t\right)\right)+\frac{\alpha }{G\left(\alpha \right)}\times \int_{0}^{t}ϒ\left(s,\Phi \left(s\right)\right)ds. \end{array}$$

Taking Equation (61) at the point *t*_*n*+1_ = (*n* + 1)*h* and *t*_*n*_ = *nh*, *n* = 0, 1, 2, 3, …, with *h* being the time step, we have

(62) $$\begin{array}{l} \begin{array}{l} \Phi \left(t_{n+1}\right)=\Phi \left(0\right)+\frac{\left(1-\alpha \right)}{G\left(\alpha \right)}ϒ\left(t_{n},\Phi \left(t_{n}\right)\right)+\frac{\alpha }{G\left(\alpha \right)}\\ \;\times \int_{t_{n}}^{t_{n+1}}ϒ\left(s,\Phi \left(s\right)\right)ds, \end{array} \end{array}$$

(63) $$\begin{array}{l} \begin{array}{l} \Phi \left(t_{n}\right)=\Phi \left(0\right)+\frac{\left(1-\alpha \right)}{G\left(\alpha \right)}\Phi \left(t_{n-1},ℵ\left(t_{n-1}\right)\right)+\frac{\alpha }{G\left(\alpha \right)}\\ \;\times \int_{t_{n}}^{t_{n+1}}ϒ\left(s,\Phi \left(s\right)\right)ds. \end{array} \end{array}$$

Taking the results of Equations (62)-(63) in

(64) $$\begin{array}{l} \begin{array}{c} \Phi \left(t_{n+1}\right)-ℵ\left(t_{n}\right)=\frac{\left(1-\alpha \right)}{G\left(\alpha \right)}\left(ϒ\left(t_{n},\Phi \left(t_{n}\right)\right)-ϒ\left(t_{n-1},\Phi \left(t_{n-1}\right)\right)\right)\\ +\frac{\alpha }{G\left(\alpha \right)}\times \int_{t_{n}}^{t_{n+1}}ϒ\left(s,\Phi \left(s\right)\right)ds, \end{array} \end{array}$$

Equation (64) in the two-step Lagrange polynomial gives

(65) $$\begin{array}{l} \begin{array}{l} \Phi \left(t_{n+1}\right)-\Phi \left(t_{n}\right)=\frac{\left(1-\alpha \right)}{G\left(\alpha \right)}\left(ϒ\left(t_{n},\Phi \left(t_{n}\right)\right)-ϒ\left(t_{n-1},\Phi \left(t_{n-1}\right)\right)\right)\\ +\frac{\alpha }{G\left(\alpha \right)}\times \int_{t_{\alpha }}^{t_{\alpha +1}}[\frac{ϒ\left(t_{n},\Phi \left(t_{n}\right)\right)}{h}\left(s-t_{n-1}\right)\\ -\frac{ϒ\left(t_{n-1},\Phi \left(t_{n-1}\right)\right)}{h}\left(s-t_{n}\right)]ds. \end{array} \end{array}$$

The aforementioned Equation (65) leads to

(66) $$\begin{array}{l} \begin{array}{l} \Phi \left(t_{n+1}\right)-\Phi \left(t_{n}\right)=\frac{\left(1-\alpha \right)}{G\left(\alpha \right)}\left(ϒ\left(t_{n},\Phi \left(t_{n}\right)\right)-ϒ\left(t_{n-1},\Phi \left(t_{n-1}\right)\right)\right)\\ +\frac{\alpha }{G\left(\alpha \right)}\times [\frac{ϒ\left(t_{n},\Phi \left(t_{n}\right)\right)}{h}\int_{t_{n}}^{t_{n+1}}\left(s-t_{n-1}\right)ds-\frac{ϒ\left(t_{n-1},\Phi \left(t_{n-1}\right)\right)}{h}\\ \int_{t_{n}}^{t_{n+1}}\left(s-t_{n}\right)ds]. \end{array} \end{array}$$

Solving the integrals in Equation (66) yields

(67) $$\begin{array}{l} \begin{array}{c} \int_{t_{n}}^{t_{n+1}}\left(s-t_{n-1}\right)ds=\frac{3}{2}h^{2},\\ \int_{t_{n}}^{t_{n+1}}\left(s-t_{n}\right)ds=\frac{1}{2}h^{2}. \end{array} \end{array}$$

Substituting Equation (67) into Equation (66), then generalizing the numerical scheme of CF is as follows:

(68) $$\begin{array}{l} \begin{array}{l} \Phi_{n+1}={ℵ}_{n}+\left[\frac{\left(1-\alpha \right)}{G\left(\alpha \right)}+\frac{3h\alpha }{2G\left(\alpha \right)}\right]\Phi \left(t_{n},\Phi_{n}\right)\\ \;-\left[\frac{\left(1-\alpha \right)}{G\left(\alpha \right)}+\frac{h\alpha }{2G\left(\alpha \right)}\right]\Phi \left(t_{n-1},{ℵ}_{n-1}\right). \end{array} \end{array}$$

Thus, in terms of our CF-fractional monkeypox transmission model with non-pharmaceutical intervention, we obtain;

(69) $$\begin{array}{l} \begin{array}{l} S_{h_{n+1}}=S_{h_{n}}+\left[\frac{\left(1-\alpha \right)}{G\left(\alpha \right)}+\frac{3h\alpha }{2G\left(\alpha \right)}\right]ϒ\left(t_{n},S_{h_{n}}\right)\\ \;-\left[\frac{\left(1-\alpha \right)}{G\left(\alpha \right)}+\frac{h\alpha }{2G\left(\alpha \right)}\right]ϒ\left(t_{n-1},S_{h_{n-1}}\right). \end{array} \end{array}$$

(70) $$\begin{array}{l} \begin{array}{l} E_{h_{n+1}}=E_{h_{n}}+\left[\frac{\left(1-\alpha \right)}{G\left(\alpha \right)}+\frac{3h\alpha }{2G\left(\alpha \right)}\right]ϒ\left(t_{n},E_{h_{n}}\right)\\ \;-\left[\frac{\left(1-\alpha \right)}{G\left(\alpha \right)}+\frac{h\alpha }{2G\left(\alpha \right)}\right]ϒ\left(t_{n-1},E_{h_{n-1}}\right). \end{array} \end{array}$$

(71) $$\begin{array}{l} \begin{array}{l} I_{h_{n+1}}=I_{h_{n}}+\left[\frac{\left(1-\alpha \right)}{G\left(\alpha \right)}+\frac{3h\alpha }{2G\left(\alpha \right)}\right]ϒ\left(t_{n},I_{h_{n}}\right)\\ \;-[\frac{\left(1-\alpha \right)}{G\left(\alpha \right)}+\frac{h\alpha }{2G\left(\alpha \right)}]ϒ\left(t_{n-1},I_{h_{n-1}}\right). \end{array} \end{array}$$

(72) $$\begin{array}{l} \begin{array}{l} Q_{h_{n+1}}=Q_{h_{n}}+\left[\frac{\left(1-\alpha \right)}{G\left(\alpha \right)}+\frac{3h\alpha }{2G\left(\alpha \right)}\right]ϒ\left(t_{n},Q_{h_{n}}\right)\\ \;-\left[\frac{\left(1-\alpha \right)}{G\left(\alpha \right)}+\frac{h\alpha }{2G\left(\alpha \right)}\right]ϒ\left(t_{n-1},Q_{h_{n-1}}\right). \end{array} \end{array}$$

(73) $$\begin{array}{l} \begin{array}{l} R_{h_{n+1}}=S_{h_{n}}+\left[\frac{\left(1-\alpha \right)}{G\left(\alpha \right)}+\frac{3h\alpha }{2G\left(\alpha \right)}\right]ϒ\left(t_{n},R_{h_{n}}\right)\\ \;-\left[\frac{\left(1-\alpha \right)}{G\left(\alpha \right)}+\frac{h\alpha }{2G\left(\alpha \right)}\right]ϒ\left(t_{n-1},R_{h_{n-1}}\right). \end{array} \end{array}$$

(74) $$\begin{array}{l} \begin{array}{l} S_{r_{n+1}}=S_{h_{n}}+\left[\frac{\left(1-\alpha \right)}{G\left(\alpha \right)}+\frac{3h\alpha }{2G\left(\alpha \right)}\right]ϒ\left(t_{n},S_{r_{n}}\right)\\ \;-\left[\frac{\left(1-\alpha \right)}{G\left(\alpha \right)}+\frac{h\alpha }{2G\left(\alpha \right)}\right]ϒ\left(t_{n-1},S_{r_{n-1}}\right). \end{array} \end{array}$$

(75) $$\begin{array}{l} \begin{array}{l} E_{r_{n+1}}=S_{h_{n}}+\left[\frac{\left(1-\alpha \right)}{G\left(\alpha \right)}+\frac{3h\alpha }{2G\left(\alpha \right)}\right]ϒ\left(t_{n},E_{r_{n}}\right)\\ \;-\left[\frac{\left(1-\alpha \right)}{G\left(\alpha \right)}+\frac{h\alpha }{2G\left(\alpha \right)}\right]ϒ\left(t_{n-1},E_{r_{n-1}}\right). \end{array} \end{array}$$

(76) $$\begin{array}{l} \begin{array}{l} I_{r_{n+1}}=S_{h_{n}}+\left[\frac{\left(1-\alpha \right)}{G\left(\alpha \right)}+\frac{3h\alpha }{2G\left(\alpha \right)}\right]ϒ\left(t_{n},I_{r_{n}}\right)\\ \;-\left[\frac{\left(1-\alpha \right)}{G\left(\alpha \right)}+\frac{h\alpha }{2G\left(\alpha \right)}\right]ϒ\left(t_{n-1},I_{r_{n-1}}\right). \end{array} \end{array}$$

## 10. Sensitivity analysis

Since an epidemiological system's parameters are either estimated or fitted, there is some degree of uncertainty in the numbers that are utilized to derive conclusions about the underlying epidemic. It is crucial to evaluate the individual effects of each parameter on the dynamics of the epidemic to identify those effects that have the greatest impact on the epidemic's spread or contraction. For biological factors included in the proposed monkeypox model, we perform the sensitivity analysis in this section. This analysis is investigated analytically by computing $\frac{\partial {ℜ}_{0}}{\partial p}$, where, *p* = (β_*hh*_, Λ_*h*_, ϕ_*h*_, μ_*h*_, γ_*h*_, ν_*h*_, and ψ_*h*_). The sensitivity of ℜ_0_ to each parameter is as follows:

$$\begin{array}{l} \begin{array}{l} \frac{\partial {ℜ}_{0}}{\partial \beta_{hh}}=\frac{\lambda_{h}\phi_{h}}{\mu_{h}\left(\gamma_{h}+\mu_{h}+\phi_{h}\right)\left(\nu_{h}+\mu_{h}+\psi_{h}\right)}>0,\\ \frac{\partial {ℜ}_{0}}{\partial \Lambda_{h}}=\frac{\beta_{hh}\phi_{h}}{\mu_{h}\left(\gamma_{h}+\mu_{h}+\phi_{h}\right)\left(\nu_{h}+\mu_{h}+\psi_{h}\right)}>0,\\ \frac{\partial {ℜ}_{0}}{\partial \phi_{h}}=\frac{\beta_{hh}\Lambda_{h}\left(\gamma_{h}+\mu_{h}\right)}{\mu_{h}\left(\gamma_{h}+\mu_{h}+\phi_{h}\right)\left(\nu_{h}+\mu_{h}+\psi_{h}\right)},\\ \frac{\partial {ℜ}_{0}}{\partial \mu_{h}}=-\frac{\begin{array}{l} \beta_{hh}\Lambda_{h}(\mu_{h}\left(\gamma_{h}+\mu_{h}+\phi_{h}\right)+\mu_{h}\left(\nu_{h}+\mu_{h}+\psi_{h}\right)\\ +\left(\gamma_{h}+\mu_{h}+\phi_{h}\right)\left(\nu_{h}+\mu_{h}+\psi_{h}\right)) \end{array}}{\mu_{h}^{2}{\left(\gamma_{h}+\mu_{h}+\phi_{h}\right)}^{2}{\left(\nu_{h}+\mu_{h}+\psi_{h}\right)}^{2}}<0,\\ \frac{\partial {ℜ}_{0}}{\partial \gamma_{h}}=-\frac{\beta_{hh}\lambda_{h}\phi_{h}}{\mu_{h}{\left(\gamma_{h}+\mu_{h}+\phi_{h}\right)}^{2}\left(\nu_{h}+\mu_{h}+\psi_{h}\right)}<0,\\ \frac{\partial {ℜ}_{0}}{\partial \nu_{h}}=-\frac{\beta_{hh}\lambda_{h}\phi_{h}}{\mu_{h}{\left(\gamma_{h}+\mu_{h}+\phi_{h}\right)}^{2}{\left(\nu_{h}+\mu_{h}+\psi_{h}\right)}^{2}}<0,\\ \frac{\partial {ℜ}_{0}}{\partial \psi_{h}}=-\frac{\beta_{hh}\lambda_{h}\phi_{h}}{\mu_{h}{\left(\gamma_{h}+\mu_{h}+\phi_{h}\right)}^{2}{\left(\nu_{h}+\mu_{h}+\psi_{h}\right)}^{2}}<0, \end{array} \end{array}$$

thus,

(77) $$\begin{array}{l} \begin{array}{l} \frac{\partial {ℜ}_{0}}{\partial \beta_{hh}}=124645.995943846\\ \frac{\partial {ℜ}_{0}}{\partial \Lambda_{h}}=16.9011519923859\\ \frac{\partial {ℜ}_{0}}{\partial \phi_{h}}=\frac{0.1746837421029776}{{\left(0.008139+\psi_{h}\right)}^{2}}\\ \frac{\partial {ℜ}_{0}}{\partial \mu_{h}}=-\frac{\begin{array}{c} 9.912\times 10^{-7}\mu_{h}^{2}+4.7035744\times 10^{-8}\mu_{h}^{2}\\ +2.797853632\times 10^{-10} \end{array}}{\begin{array}{c} \mu_{h}^{2}(\mu_{h}^{4}+0.014236\mu_{h}^{3}+0.00012055158688\mu_{1}\\ +7.17083788864\times 10^{-7}) \end{array}},\\ \frac{\partial {ℜ}_{0}}{\partial \gamma_{h}}=-\frac{0.15096125860751}{{\left(\gamma_{h}+0.007039\right)}^{2}},\\ \frac{\partial {ℜ}_{0}}{\partial \nu_{h}}=-\frac{0.59600691709814}{{\left(\nu_{h}+0.056039\right)}^{2}},\\ \frac{\partial {ℜ}_{0}}{\partial \psi_{h}}=-\frac{0.59600691709814}{{\left(\nu_{h}+0.056039\right)}^{2}}. \end{array} \end{array}$$

The sensitivity index technique will help measure the most sensitive parameters for the fundamental reproductive number ℜ_0_ (Borgonovo et al. (51) for details about the method). The fundamental reproduction number's normalized sensitivity index is provided by $S_{p}^{{ℜ}_{0}}=\frac{\partial {ℜ}_{0}}{\partial p}.\frac{p}{{ℜ}_{0}}$, where p is a parameter as defined earlier. We obtain

$$\begin{array}{l} \begin{array}{l} S_{\beta_{hh}}^{{ℜ}_{0}}=1,\\ S_{\Lambda_{h}}^{{ℜ}_{0}}=1,\\ S_{\phi_{h}}^{{ℜ}_{0}}=\frac{\gamma_{h}+\mu_{h}}{\gamma_{h}+\mu_{h}+\phi_{h}},\\ S_{\mu_{h}}^{{ℜ}_{0}}=\frac{\begin{array}{c} \mu_{h}\left(\gamma_{h}+\mu_{h}+\phi_{h}\right)+\mu_{h}\left(\nu_{h}+\mu_{h}+\psi_{h}\right)\\ +\left(\gamma_{h}+\mu_{h}+\phi_{h}\right)\left(\nu_{h}+\mu_{h}+\psi_{h}\right) \end{array}}{\left(\gamma_{h}+\mu_{h}+\phi_{h}\right)\left(\nu_{h}+\mu_{h}+\psi_{h}\right)}, \end{array} \end{array}$$

$$\begin{array}{l} \begin{array}{l} S_{\gamma_{h}}^{{ℜ}_{0}}=\frac{\gamma_{h}}{\gamma_{h}+\mu_{h}+\phi_{h}},\\ S_{\nu_{h}}^{{ℜ}_{0}}=\frac{\nu_{h}}{\nu_{h}+\mu_{h}+\psi_{h}},\\ S_{\psi_{h}}^{{ℜ}_{0}}=\frac{\psi_{h}}{\nu_{h}+\mu_{h}+\psi_{h}}. \end{array} \end{array}$$

**Figure 3:**
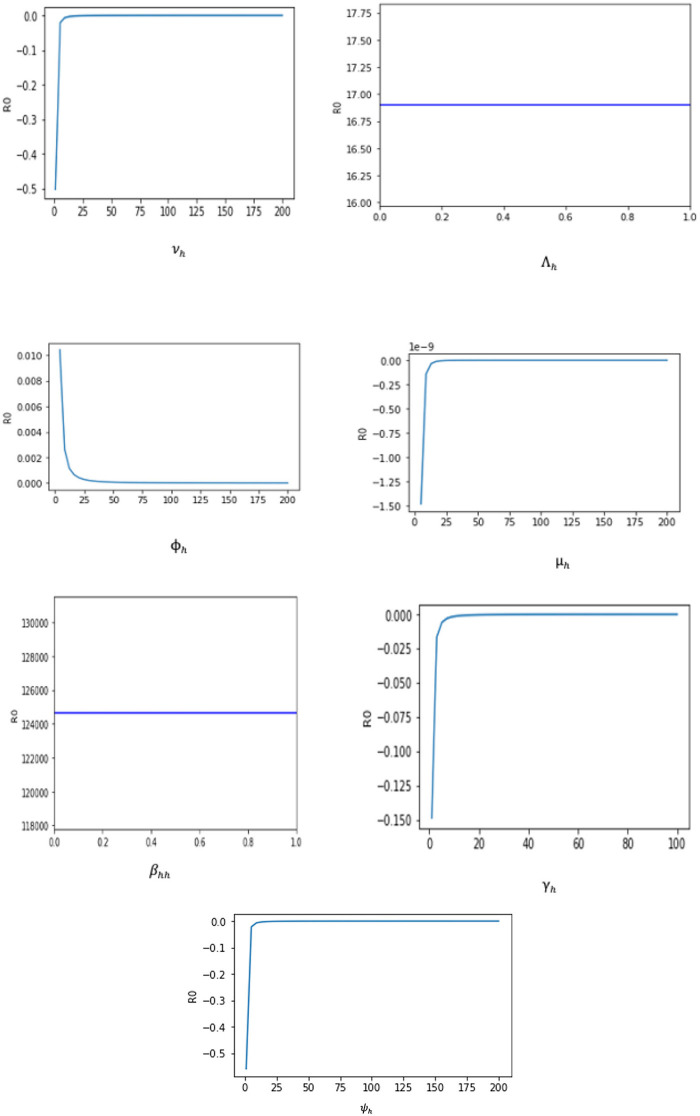
The sensitivity analysis of ℜ_0_ with respect to the parameter p of the system (1).

The sensitivity indices using the parameter values given in Table 2 are presented in Table 3. The sensitivity analysis of β_*hh*_, Λ_*h*_, ϕ_*h*_, ψ_*h*_, ν_*h*_, γ_*h*_, and μ_*h*_ with respect to ℜ_0_ and their graphs are presented in Figure 3.

**Table 3:** The sensitivity index of ℜ_0_ with respect to parameter p of the system (1).

**Parameter**	**Sensitivity index**
ν_*h*_	–0.0014
Λ_*h*_	1
ϕ_*h*_	–1.003
μ_*h*_	–1.003
β_*hh*_	1
γ_*h*_	–0.5350
ψ_*h*_	–0.9979

Two of the sensitivity indices are positive while others are negative, as can be seen in Table 3. Additionally, the majority of these indices are functions of the Caputo–Fabrizio fractional monkeypox model parameters. This implies that changing one of the parameters slightly will alter the dynamics of the epidemic. The basic reproductive number ℜ_0_ normalized sensitivity indices to the Caputo–Fabrizio fractional monkeypox model parameters are calculated. We conclude that increasing the rate of recovery and the rate of identifying suspected cases, that is, isolation and quarantining of the monkeypox virus carrier will aid in decreasing the ℜ_0_, which is an affirmation of the effect of non-pharmaceutical intervention to combat the spread of the virus.

## 11. Discussion and conclusion

**Figure 4:**
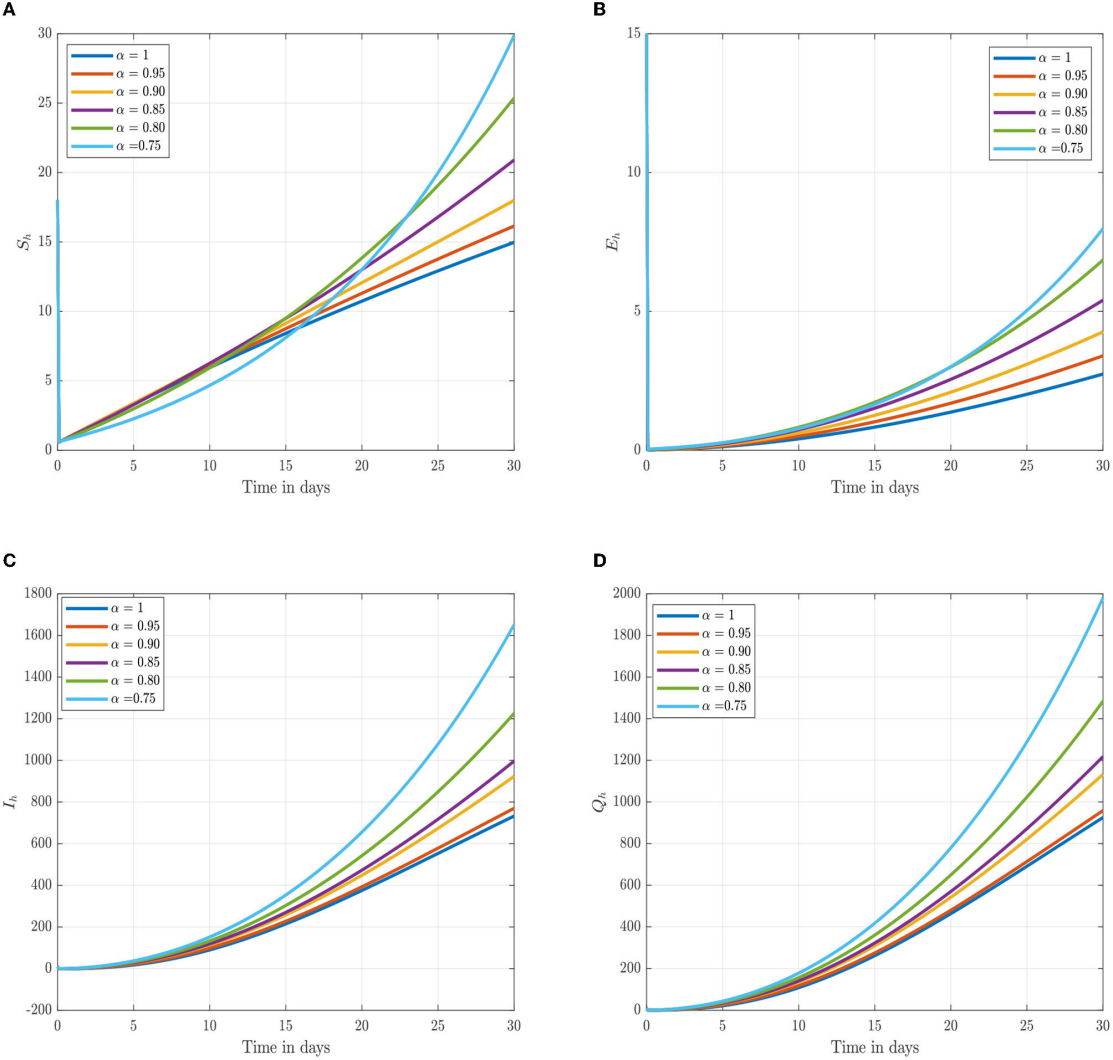
Numerical trajectory of the CF-fractional-order derivative, α, of Equation (2). **(A)** Dynamics of susceptible (*S*_*h*_) humans class. **(B)** Dynamics of expose (*E*_*h*_) humans class. **(C)** Dynamics of infected (*I*_*h*_) humans class. **(D)** Dynamics of quarantine (*Q*_*h*_) class.

**Figure 5:**
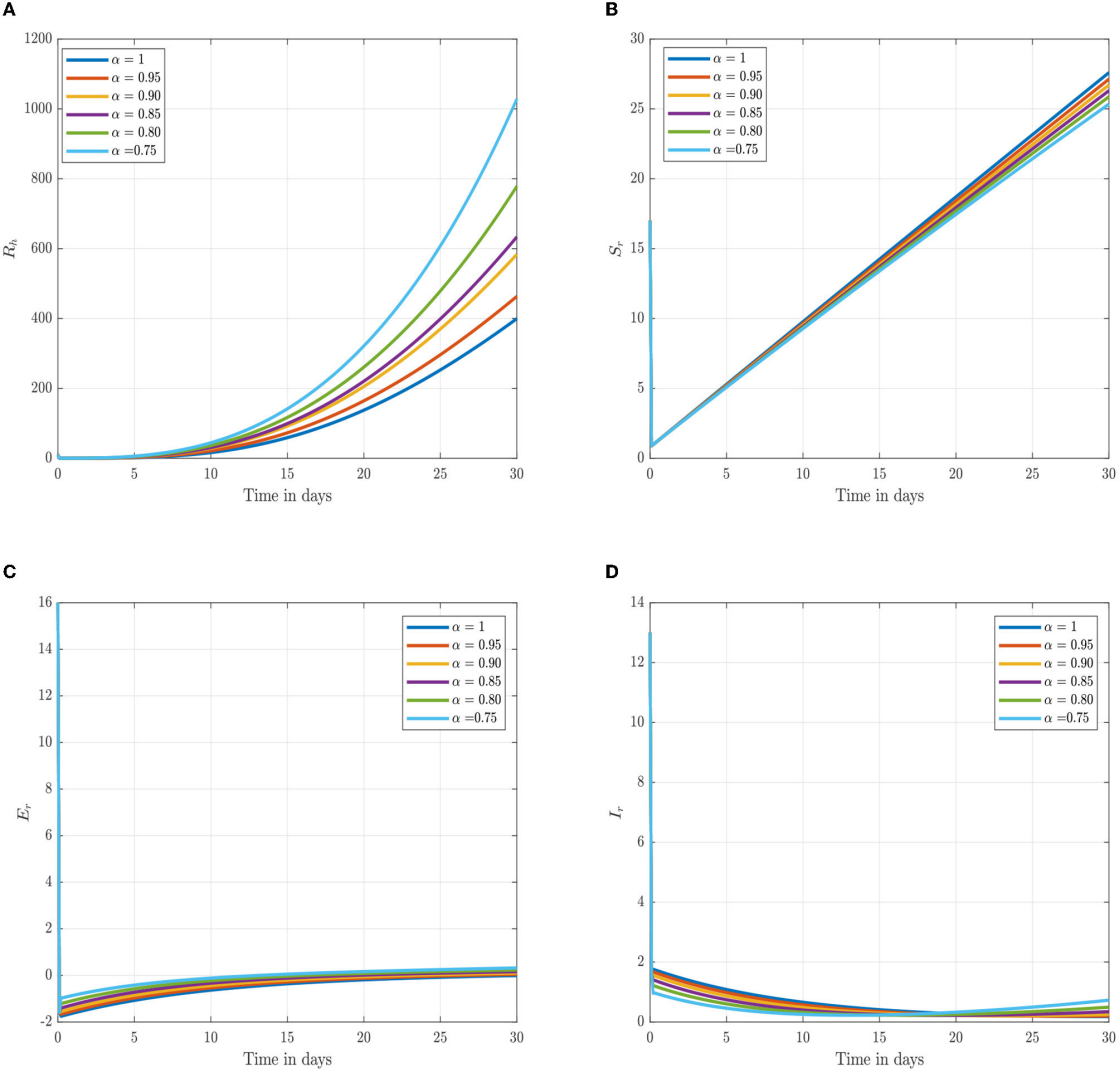
Numerical trajectory of the CF-fractional-order derivative, α, of Equation (2). **(A)** Dynamics of recovery (*R*_*h*_) class. **(B)** Dynamics of susceptible (*S*_*r*_) rodents class. **(C)** Dynamics of exposed (*E*_*r*_) rodents class. **(D)** Dynamics of asymptomatic infected (*I*_*r*_) rodents class.

**Figure 6:**
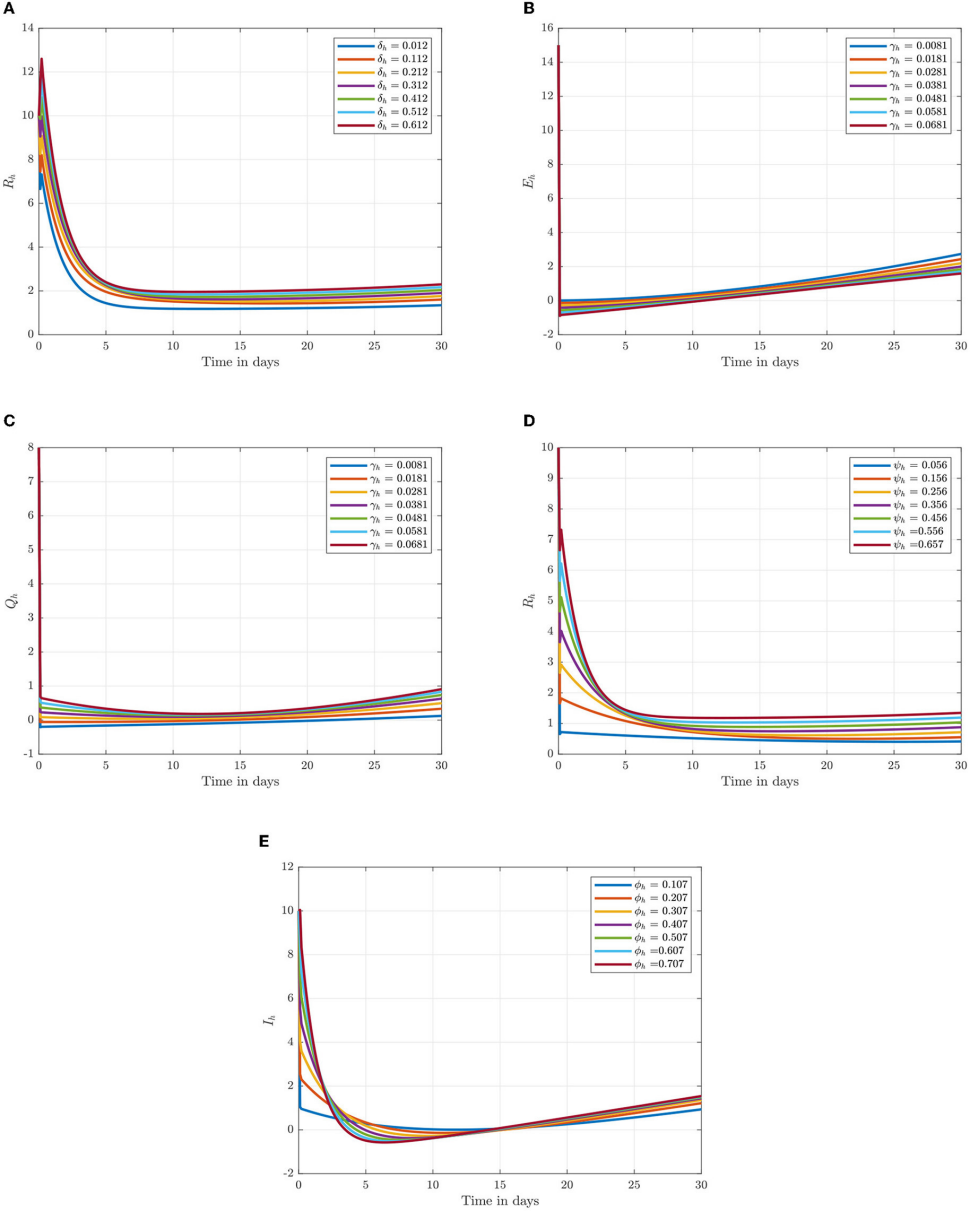
Numerical trajectory of varying δ_*h*_, γ_*h*_, ψ_*h*_, and ϕ_*h*_ when α = 0.95. **(A)** Variation of δ_*h*_ on recovery class. **(B)** Variation of γ_*h*_ on expose class. **(C)** Variation of γ_*h*_ on quarantine class. **(D)** Variation of ψ_*h*_ on recovery class. **(E)** Variation of ϕ_*h*_ on infected class.

Following the estimation of parameter values and data fitting, we simulate the Caputo–Fabrizio fractional monkeypox virus model using the parameter values, as presented in Table 2. The fitted Caputo–Fabrizio curve and ℜ_0_ are given in Figure 2. Figures 4, 5 show dynamic behavior for all the nine compartments involved in the proposed Caputo–Fabrizio fractional monkeypox virus model. We observed a significantly high susceptibility and infection in the solution pathways of individual species. The work of Hammouch et al. (52), Bonyah et al. (53), Peter (54), and Sene (55) have provided a strong basis for the discussion of our results. This indicates that, whenever the memory index increases, the rate at which people get infected with monkeypox virus reduces and vice versa, which then indicates that, using fractional order, we can obtain clear qualitative information on monkeypox virus transmission. In Figure 6, we varied the input parameter γ_*h*_ on quarantine and exposed, respectively, to observe variation in the system dynamics. We noticed the contribution of this parameter in the transmission pathways of infected individuals. In a similar way, we varied the input parameters δ_*h*_ and ψ_*h*_ on individual recovery and noticed the variation in the trajectory of monkeypox recovery. We discovered that the rate at which humans and rodents move from exposed to infectious stage is also important and potentially dangerous in terms of increasing the level of monkeypox infection.

In conclusion, we provided a brief overview of the monkeypox virus and the dynamics of its transmission in this study. We investigated the spread of monkeypox virus and its effect on non-pharmaceutical intervention, thus quarantine. Positiveness, invariance, boundedness, and equilibrium points of the solutions are thus examined as fundamental features of mathematical models. We considered real data of the monkeypox virus from the United Kingdom, and the best fit curve has been obtained (see Figure 2). As a result, we created a novel, dimensionally consistent Caputo–Fabrizio fractional-order model. Krasnoselskii's fixed point theorem has been used to demonstrate that the system has a solution. The Adams–Bashforth method has been used to display numerical simulations of the suggested pandemic model for various fractional orders and parameter values. We looked into the impact of factors on the expansion and contraction of the quarantine compartment, recovery compartment, and infected compartment on the spread and regression of the pandemic with the use of numerical simulations. As can be inferred from the data, it is clear that the fractional-order equations can help explain this unique effect of the monkeypox. Real-world data can be used to test the accuracy of a mathematical model that has been created. The key challenge, however, is where to find these data and/or how to obtain the right curve for the collected data. The mathematical representation of the monkeypox has been the subject of numerous studies. To the best of our knowledge, there is still no research on fractional modeling that uses actual data on the monkeypox in the United Kingdom. Using actual data on the monkeypox from the United Kingdom, a fractional-order modeling has been shown in this study. The numerical results of this study show that the spread of monkeypox can be stopped if the number of contacts with infected people can be decreased through methods such as effective mass education, improved quarantine facilities, or increased testing of the general population, that is, performing routine tests not only on exposed individuals but also on those who have come into contact with infected patients. As a result, these studies offer other professionals and scientists who focus on infectious diseases insight that may help them in future to control the outbreak of monkeypox and contribute to the development of further treatment options. This study may provide insight into potential future research projects in this regard. Future study of the monkeypox can take into account other fractional operator types, both with and without single kernels. Furthermore, data imputation techniques can be used to fit rodent population parameters from the number of monkeypox disease since the number of rodents cannot be determined.

## Data availability statement

The original contributions presented in the study are included in the article/supplementary material, further inquiries can be directed to the corresponding author.

## Author contributions

KO, EA, and MN: conceptualization. EA, AA, UA, and KO: methodology, software, and formal analysis. EA, UA, and KO: validation, investigation, and visualization. MN and AA: resources. EA, MN, UA, and KO: data curation. EA and KO: writing and original draft preparation, writing, reviewing, and editing. KO and MN: supervision and project administration. All authors have read and agreed to the published version of the manuscript.
